# Complex ecological and socioeconomic impacts on medicinal plant diversity

**DOI:** 10.3389/fphar.2022.979890

**Published:** 2022-10-19

**Authors:** Ming-Xu Zhang, Yuan Chen, Jing-Xia Guo, Ru Zhang, Ya-Qiong Bi, Xin-Xin Wei, Hui Niu, Chun-Hong Zhang, Min-Hui Li

**Affiliations:** ^1^ Inner Mongolia Hospital of Traditional Chinese Medicine, Hohhot, China; ^2^ Inner Mongolia Institute of Traditional Chinese and Mongolian Medicine, Hohhot, China; ^3^ Baotou Medical College, Baotou, China; ^4^ Inner Mongolia Medical University, Hohhot, China; ^5^ Inner Mongolia University, Hohhot, China; ^6^ Inner Mongolia Key Laboratory of Characteristic Geoherbs Resources Protection and Utilization, Baotou, China

**Keywords:** medicinal plants diversity, species distribution model, spatial analysis, generalized additive model, socioeconomic development

## Abstract

Medicinal plant diversity (MPD) is an important component of plant diversity. Over-collection based on medicinal and economic value has the potential to damage the stability of the regional ecosystem. It is important to understand the current distribution of MPD and the factors influencing it. However, it is still unclear whether environmental and socioeconomic conditions have an impact on their distribution. We selected the Inner Mongolia as a representative study area which covers a wide area, accounting for 12.29% of China’s national land area and 0.79% of the world’s land area. At the same time, the region is a long-standing traditional medicinal area for Mongolians in China. Therefore, the region is significantly influenced by changes in environmental factors and socio-economic factors. We used 9-years field survey of the distribution of medicinal plants in Inner Mongolia for assessing the distribution of MPD as influenced by environmental and socioeconomic activities by combining spatial analyses, species distribution models, and generalized additive models. The results from the spatial analysis show that the western region of Inner Mongolia is the main cold spot area of the MPD, and the central-eastern and northeastern regions of Inner Mongolia are the main hot spot areas of the MPD. At the same time, the distribution of cold spots and hot spots of MPD is more obvious at large spatial scales, and with the refinement of spatial scales, the cold spots in scattered areas are gradually revealed, which is indicative for the conservation and development of MPD at different spatial scales. Under the future climate change of shared socioeconomic pathways (SSP), areas with high habitat suitability for medicinal plants remain mainly dominated by the Yellow River, Yin Mountains, and Greater Khingan Range. Notably, the SSP245 development pathway remains the most significant concern in either long- or short-term development. The nonlinear relationship between the driving factors of MPD at different spatial scales shows that temperature, precipitation and socioeconomic development do have complex effects on MPD. The presence of a certain temperature, altitude, and precipitation range has an optimal facilitation effect on MPD, rather than a single facilitation effect. This complex nonlinear correlation provides a reference for further studies on plant diversity and sustainable development and management. In this study, the spatial distribution of medicinal plant resources and the extent to which they are driven by ecological and socioeconomic factors were analyzed through a macroscopic approach. This provides a reference for larger-scale studies on the environmental and socioeconomic influences on the distribution of plant resources.

## 1 Introduction

Medicinal plant diversity (MPD) is one of the key components of plant diversity, and medicinal plants are one of the material bases of our present medical and food supply. MPD can provide multiple benefits to humans in terms of ecosystem function and resilience to climate change and other disturbances (García-Palacios et al., 2018; Ebert and Engels, 2020). However, the productive use and consumption of these materials are also driving forces behind the loss of plant diversity, which in turn affects ecosystem functioning and has socioeconomic impacts worldwide (Crenna et al., 2020). Therefore, ensuring the controlled collection of medicinal plants through sustainable policy instruments plays an important role in areas where traditional medicine is predominantly used.

With the fourth national survey of Chinese medicine resources in China, there is a growing concern about the status of medicinal plant resource (Huang, 2018). The complex climate change and socioeconomic conditions in Inner Mongolia have important impacts on the diversity of medicinal plants and deserve to be studied as a representative region. On the one hand, Inner Mongolia is a vast area, accounting for 12.29% of China’s land area and 0.79% of the global land area. Its wide longitudinal span has resulted in rich topographic variations, providing geographic conditions for the flora variation (Zhao et al., 2020). On the other hand, the region has a long history of Mongolian medicinal applications as a major gathering place of Mongolians. This has led to a very wide application of local medicinal plants. Therefore, the Inner Mongolia region is representative of the research on the diversity of medicinal plants. At the same time, it is important to establish a method for quantifying regional locations in terms of diversity (hotspots areas) and habitat suitability, and to link the state of knowledge of diversity to inter-regional governance tools for further conservation and use (Soto-Navarro et al., 2021; Urcádiz-Cázares et al., 2021).

Firstly, this study computationally and visually represents the study area datasets at different spatial scales through spatial analysis of Geographic Information System (GIS). This GIS-based collection and analysis of data can provide an integrated approach to MPD data monitoring at the local, regional and global levels. (Alkoy et al., 2007; VoPham et al., 2015). Based on the narrow and wide territory of Inner Mongolia, the entire study area was divided into 50, 100, 150, and 200 km resolution grids for analysis. Such small-scale measurements can more accurately identify the geographical extent of the diversity hotspots in the region (Xia et al., 2022). This study aimed to provide better guidance for local management authorities and to conduct a study of diversity distribution patterns based on administrative areas. Therefore, this study also analyzed MPD in Inner Mongolia on a county-by-county basis. Secondly, Species distribution models are effective tools for predicting the habitat suitability of target species that have been widely used in conservation. It was used to link the environment to MPD under current and future climates, and to explore the impact of the environment on MPD. Finally, considering the complexity of socioeconomic factors, generalized additive model (GAM) was used to explore the nonlinear relationship between MPD and socioeconomic factors. It can guide management in making choices about the balance between ecological protection and economic development. Based on our 9 years (December 2012—September 2021) field survey on the distribution status of medicinal plant resources, this study aims to combine multidisciplinary approaches to establish a comprehensive evaluation of ecological and socio-economic impacts on MPD and provide research avenues for diversity conservation in Inner Mongolia and globally. It explores MPD of the diverse areas from different spatial scales and spatial and temporal dimensions and provides guidelines for local governments for responding to international policies on sustainable development and diversity conservation by combining non-linear analysis of ecological and socioeconomic factors.

## 2 Materials and methods

### 2.1 Overview of the study area

Inner Mongolia is located at 97°10′18″-115°31′14″ E and 47°05′53″-37°24′26″ N, in the north of China, extending from the northeast to the southwest, in a long and narrow shape, stretching from east to west for 2,400 km, covering an area of 1.183 million km. Inner Mongolia has a continental monsoon climate, with complex and diverse environmental changes (Zhang et al., 2021). The terrain changes from west to east, with many mountain ranges such as the Greater Khingan Range and the Yin Mountain. Simultaneously, many rivers such as Heilongjiang, Liaohe, and Yellow River flow through the territory (Huang, 2018). Inner Mongolia is located in the temperate semi-arid and arid region of the Asian continent, so grassland vegetation is the main vegetation type in the region, but its desert vegetation is also relatively developed. At the same time, forest vegetation is mainly distributed in the sub-humid and humid areas in the east. The complex geographical environment and rich vegetation flora provide a certain foundation for the formation of medicinal plant diversity in this area (Chinese Academy of Sciences Inner Mongolia Ningxia Comprehensive Expedition Team., 1985).

### 2.2 Data collection

**FIGURE 1:**
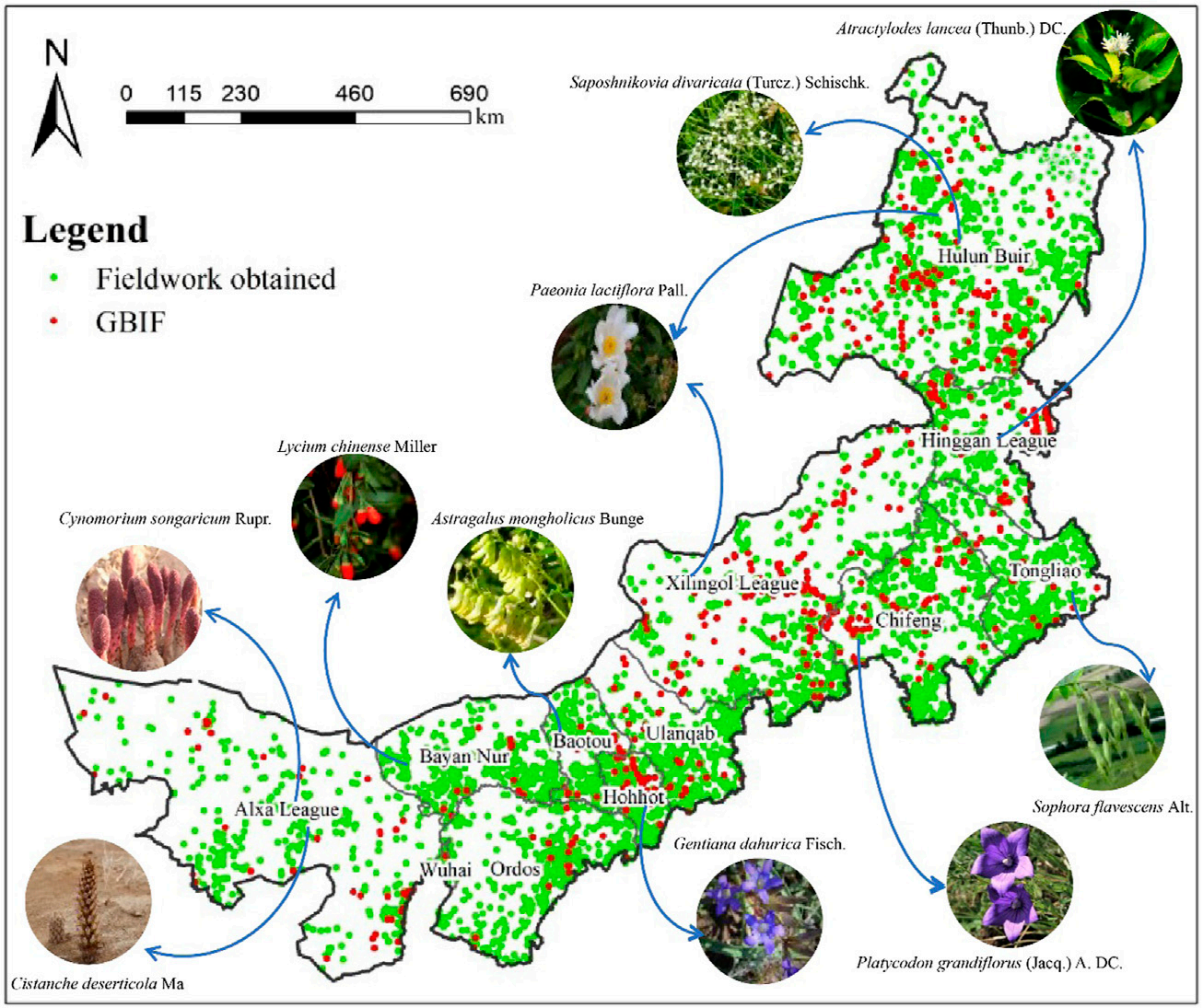
Sampling points of the distribution of medicinal plants in Inner Mongolia (The plants in the picture have a relatively wide distribution in various places. Global Biodiversity Information Facility (GBIF) database is an international network and data infrastructure funded by governments worldwide and aims to provide open access to data about all types of life on Earth. The distribution data of medicinal plants with distribution in Inner Mongolia were screened from this data).

In this study, the survey program was developed in 2012, and the detailed distribution information of medicinal plants in 103 counties in Inner Mongolia was recorded through fieldwork in 9-years (December 2012—September 2021), using counties as the basic survey unit. The specific methodology is detailed in Supplementary Materials. Nevertheless, the possibility of sampling bias remains, as the complex topography of Inner Mongolia means that many areas may be inaccessible. We supplemented this with recorded plant data from the Global Biodiversity Information Facility (GBIF) database. GBIF is an international network and data infrastructure funded by governments worldwide and aims to provide open access to data about all types of life on Earth. The plant occurrence data of the GBIF database are derived from many sources, including everything from museum specimens collected in the 18th and 19th centuries to geotagged smartphone photos shared by amateur naturalists in recent days and weeks. This makes data collection more meaningful. Finally, we used the 51,180 points data of 2,194 species obtained from the Inner Mongolia census and the 1,044 points data of 687 species supplemented by the GBIF database. Simultaneously, we performed operations such as the elimination of duplicate data. The final distribution of all sampling points is shown in Figure 1.

### 2.3 Global autocorrelation analysis

Global autocorrelation analysis (Global Moran’s *I* analysis index) can effectively identify spatial correlations (Upton and Fingleton, 1985; Boots and Getis, 1988). The value range of Moran’s I was (-1,1). The standardized statistics Z and *p* were used to test the applicability of the index. When, *p* < 0.1, *Z* > 1.65, and 0 < Moran’s *I* < 1, it means that the types of medicinal plant resources in Inner Mongolia have a positive correlation with the spatial distribution (Zhang and McGrath, 2004). It is in the form of spatial aggregation distribution; when *p* < 0.1, *Z* > 1.65, and 1 < Moran’s *I* < 0, it means that the types of medicinal plant resources in Inner Mongolia have a negative correlation in spatial distribution and are in the form of spatial discrete distribution; if *p* > 0.1, or Moran’s *I* = 0, -1. 65 < *Z* < 1. 65 means that the types of medicinal plant resources in Inner Mongolia are not related to spatial distribution and are distributed randomly in space (Jaya et al., 2019). The spatial weights were row normalized during the spatial calculations to prevent the possibility of global Moran’s I index being outside (-1-1) (Bivand and Wong 2018).

Moran’s *I* is calculated as follows:

$$I=\frac{n}{S_{0}}\frac{\overset{n}{\underset{i=1}{\sum }}\overset{n}{\underset{j=1}{\sum }}w_{i,j}z_{i}z_{j}}{\overset{n}{\underset{i=1}{\sum }}z_{i}^{2}},$$ (1)

Where n is the number of observations of the whole region, $z_{i}$ is the value of the variable at a particular position minus the average of all positions, $z_{j}$ is the value of the variable at another specific position minus the average of all positions, $w_{i,j}$ is a spatial weight between locations of i relative to j. And $S_{0}$ is the aggregation of all spatial weights, and is calculated as follows:

$$S_{0}=\overset{n}{\underset{i=1}{\sum }}\overset{n}{\underset{j=1}{\sum }}w_{i,j},$$ (2)

$$Z=\frac{I-E\left[I\right]}{\sqrt{V\left[I\right]}},$$ (3)

and:

E[I]=−1/(n−1), (4)

$$V\left[I\right]=E\left[I^{2}\right]-E{\left[I\right]}^{2}.$$ (5)

### 2.4 Local Moran’s *I* analysis

Decomposition of the global spatial autocorrelation index can indicate the degree of difference and significance between regions. The local Moran’s I analysis measures the degree of difference between a region and its neighboring region and its significance and is a decomposition of the global Moran’s *I* (Anselin 1995). The magnitude of the local Moran’s *I* is a better visual comparison of the magnitude of diversity differences between value regions (Liu et al., 2018). However, this method of analysis does not allow for the representation of specific geographical areas at the level of significance.

### 2.5 Hotspot analysis

Hotspots analysis method is used to identify the high-value or low-value spatial clustering of attribute elements for determining whether each regional unit belongs to the spatial distribution pattern of high-value or low-value clusters (Getis and Ord, 1992). With the G index calculation method developed by Getis and Ord, it is possible to determine whether there is clustering within the dataset, as well as the degree of clustering above or below the mean and the concentration of local clusters (Getis and Ord, 1996). The obtained z-score and *p*-value allow us to determine where the clustering of high- or low-value elements occurs in space. Hotspot elements that become statistically significant should have high values and are surrounded by other elements that also have high values. In addition, the local sum of elements and their neighboring elements is compared with the sum of all elements (Getis and Ord 1992). If the G index is positive and significant, it indicates that the medicinal plant resources around the area are rich in variety and tend to be spatially agglomerated, belonging to high-value spatial agglomerations; if the G index is negative and significant, it indicates that the area is used for medicinal purposes plant resources experience species scarcity and tend to accumulate in space (Laffan, 2006; Mueller-Warrant et al., 2008).

### 2.6 Maxent’s parameter settings

Maxent is a machine-learning algorithm that uses presence-only data to determine the predicted suitability of local conditions for a given species (Phillips et al., 2006). Maxent model has been used in previous studies to assess the distribution of species (Xu et al., 2021; Yue et al., 2022; Zhu et al., 2022). This study aimed to explore the distribution of species screened under different conditions in Inner Mongolia. Therefore, we did not use the traditional knife-cut method or response curves to assess ecological impacts (Phillips et al., 2006). Maxent is carried out in the GUI of maxent and it was set as follows: the maximum number of iterations was 1 million, the convergence threshold was 0.0005, and the random test ratio was set to 10; i.e., 90% of the point data were randomly selected as training data, and the remaining 10% of data points were test data. Cross-validation (dividing the dataset into 10 copies, of which 9 copies were used as training data and 1 copy was used as test data for the experiment in turn) was used as the replication run type, and the maximum number of background points and the remaining parameters were set as default (Elith et al., 2011). After calculating the results for each species in the ASC format in ArcGIS10.2 (ESRI Inc., Redlands, CA, United States), the results were converted to tiff format and the resulting maps for each species were overlaid. To ensure the accuracy of the model predictions, we considered the predicted results for each species to ensure that the AUC values were all greater than 0.7 (Parichehreh et al., 2020). In the traditional logistic output model, the resultant values for each species are in the range of 0–1, which approximates the probability of the distribution of the species. We then selected data from three taxa for analysis: 1) species with more than 200 sampling points, 2) species with between 10 and 30 sampling points in Inner Mongolia, and 3) plants recorded according to the plant protection list. They represent the distribution of widely distributed or less distributed species and endangered protected species in Inner Mongolia, respectively.

### 2.7 Selection of environmental variables for modeling

The current and future climate data, including 19 bioclimatic variables (Table 1), were downloaded from the World Climate Database, with a resolution of 2.5 arcmins (Liu et al., 2019). In this study, the BCC-CSM-MR model (Beijing Climate Center Climate System Model) with strong simulation capability in China was selected as the future climate scenario (Feng et al., 2021). The BCC-CSM-MR model published in the World Climate Research Program Coupled Model Intercomparison Project (CMIP6) contains four shared socioeconomic pathways (SSPs), green-growth paradigm (SSP126), middle-of-the-road development along historical pattern (SSP245), regionally-heterogeneous development (SSP370), and development path dominated by high energy demand supplied by fossil-fuel (SSP585) (Zeng et al., 2020; Xu et al., 2021). Each scenario was divided into two future periods: the 2030s (2021–2070), the 2070s (2060–2080). The elevation data were derived from SRTM elevation data (Fick and Hijmans 2017). Given the large number of species in this study, we used the most widely accepted data (many studies used these data as the most basic spatial variables), and added some influences related to their biological properties depending on the plant.

**TABLE 1:** The environment variables information.

Abbreviation	Environment variables	Unit
Bio_1	Annual mean temperature	◦C
Bio_2	Mean diurnal range	◦C
Bio_3	Isothermality	
Bio_4	Temperature seasonality	◦C
Bio_5	Max temperature of warmest month	◦C
Bio_6	Min temperature of coldest month	◦C
Bio_7	Temperature annual range	◦C
Bio_8	Mean temperature of wettest quarter	◦C
Bio_9	Mean temperature of driest quarter	◦C
Bio_10	Mean temperature of warmest quarter	◦C
Bio_11	Mean temperature of coldest quarter	◦C
Bio_12	Annual precipitation	mm
Bio_13	Precipitation of wettest month	mm
Bio_14	Precipitation of driest month	mm
Bio_15	Precipitation seasonality	
Bio_16	Precipitation of wettest quarter	mm
Bio_17	Precipitation of driest quarter	mm
Bio_18	Precipitation of warmest quarter	mm
Bio_19	Precipitation of coldest quarter	mm
Elv	Elevation	m
Zblx	Vegetable type	
Trlx	Soil type	

### 2.8 Effects of different drivers on the MPD

The general additive model is receiving increasing attention from scholars, and GAM has good flexibility, nonlinearity, and smoothing capability for spatial data (Denis et al., 2002). The parameters estimated in the model were in the form of smooth-functions which constructs nonlinear functions through functions that are continuously derivable of infinite order in their domain of definition (Wood, 2013). The code optimization in R language in this study ensures that the model effective degree of freedom (EDF) is below 0.75 × k and does not change significantly when k increases. The final confirmation was *k* = 3 for each variable which can effectively prevent over-fitting of the model and model stability (Zhang et al., 2015). We used the GAM function of the MGCV package in R 4.1.1 to implement the evaluation and analysis of the generalized summation model (Wood, 2011). Because of the different underlying analysis units, we divided the drivers into two parts: one part was the ecological environmental-climate data under different spatial grids and the other part considers the data on human social development under administrative divisions (including municipal and county levels). Because of the very large amount of data collected, to prevent the influence of these factors from being difficult to explain, we conducted Principal Components (PC) analysis on the two parts of data separately to achieve the purpose of data dimensionality reduction. The ecological data used in this study were mainly climatic data used in the species distribution model analysis, and simultaneously, data based on soil type and vegetation type were added. The 25 human social development data used in this study were obtained from the population census, the Inner Mongolia Bureau of Statistics, and its published statistical yearbooks (Supplementary Table S5).

## 3 Results

### 3.1 Global autocorrelation analysis

First, a global autocorrelation analysis was conducted on the collected medicinal plant distribution data for confirming the spatial distribution relationships of MPD. Under the assumption of normal distribution, MPD at different spatial scales and different administrative districts in Inner Mongolia shows a positive correlation distribution in spatial distribution, which is a highly significant clustered distribution (Supplementary Table S1). Therefore, this clustered spatial distribution pattern indicates the existence of hotspots of medicinal plant resources in Inner Mongolia and the necessity of further spatial analysis for exploring their specific geographical locations.

### 3.2 Local Moran’s I analysis

**FIGURE 2:**
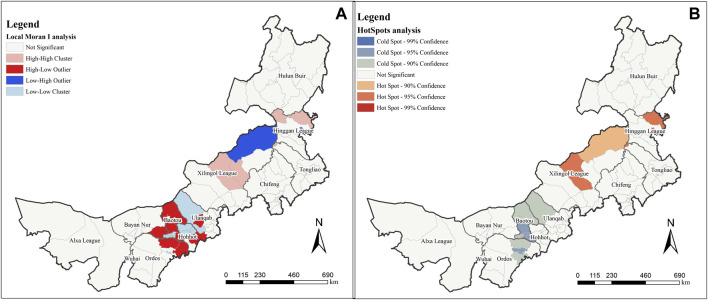
Spatial analysis under county administrative division. **(A)** is Local Moran’s *I* analysis result; **(B)** is HotSpots analysis.

Using Local Moran’s I analysis, this study analyzed the spatial outliers of MPD at county-level administrative districts and different spatial scales (Figure 2A). When using counties and districts as research units, several districts in the urban areas of Hohhot and Baotou are L-L clusters or insignificant, while the surrounding urban suburbs around their radiation (including parts of Ordos and Bayan Nur) are H-L Outliers. In addition, the H-H clusters in the central part of Xilingol League was compared with the surrounding H-L outlier, and the MPD in the central region was abnormally rich compared with the surrounding regions. In our survey, we found that Ordos, Hohhot, and Baotou were the three cities with the best economic development in Inner Mongolia. Therefore, the abnormal distribution of MPD in this region may be related to its developed urban areas, and a high level of urbanization may harm MPD in this region. These three cities are mainly located at the foot of the Yinshan Mountains, such as Guyang County, Baotou City, and Wuchuan County, Hohhot City, which have extensive planting industries in their suburban areas, which may also be one of the factors that threaten the diversity of medicinal plants in the suburban areas.

**FIGURE 3:**
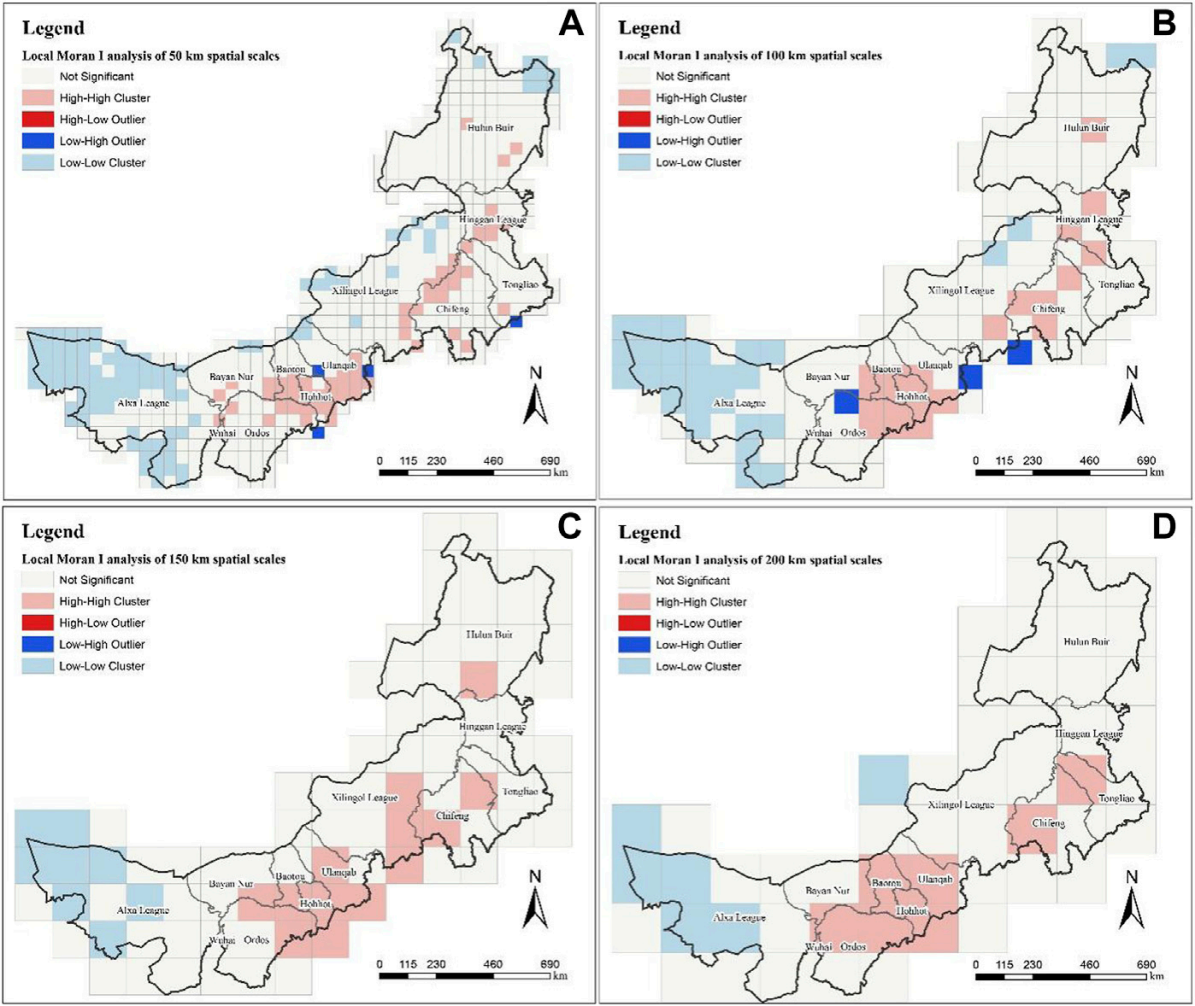
Local Moran’s *I* analysis at different scales. **(A)** is the result at the spatial scale of 50 km; **(B)** is 100 km; **(C)** is 150 km; **(D)** is 200 km.

At different spatial scales, the H-H clusters are all concentrated in the line from Hulun Buir in the northeast of Inner Mongolia to Wuhai City in the southwest of Inner Mongolia, and the L-L clusters are all concentrated in the Alxa League. Although there are differences in the spatial distribution of large-scale (150 km and 200 km) MPD, the aggregation of MPD from a small scale is still different. On a large spatial scale of 200 km (Figure 3D), it can be seen that the H-H clusters is more concentrated, mainly in the north of Tongliao City, Chifeng City, Baotou City, Ulanqab City, Hohhot City, Ordos City and Wuhai City. At the spatial scale of 150 km (Figure 3C) and smaller spatial scales, new H-H clusters appeared in Hulun Buir, and L-L clusters appeared scattered in the large-scale H-H clusters. In addition, although there is no spatial distribution of H-L outlier at large spatial scales, there are scattered distributions at spatial scales of 100 km (Figure 3B) and 50 km (Figure 3A), which means that different spatial scales can indicate the aggregation degree of different medicinal plant diversity in a unified area. In other words, with the further refinement of the spatial scale, the large-scale high-value clusters are gradually subdivided into some low-value regions, and the regions that are not significantly clustered will also have some low-value clusters. This has important guiding significance for the refined understanding of the resource aggregation degree of medicinal plant diversity.

#### 3.3 Hot spots analysis

For viewing the spatial distribution of MPD in terms of significance, we simultaneously performed a Hot Spot analysis. The results of the MPD based on county-level administrative directives are shown in Figure 2B. At different significance levels, the hot spot aggregations are different to the areas in the local Moran’s I analysis, with the northeastern Hinggan League and central Xilingol League being hot spot aggregation regions (95% confidence). Central and southern Baotou and a small area in eastern Ordos are cold spot aggregation regions (95% confidence). The northeastern Xilingol league is a hot spot aggregation region while northern Baotou, northern Ulanqab, and parts of eastern Ordos are cold spot aggregation regions (90% confidence). No region passed the 99% confidence level.

**FIGURE 4:**
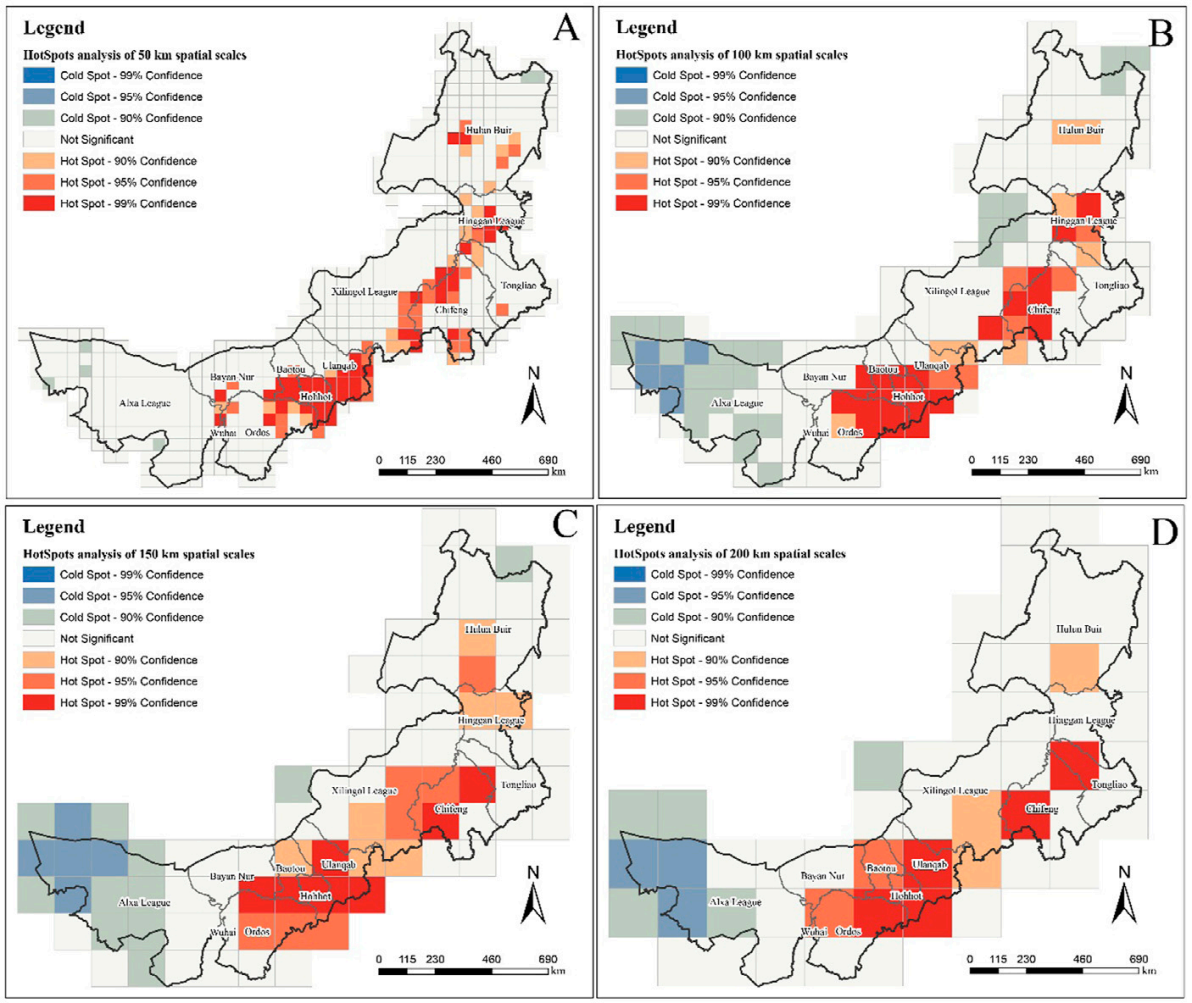
Hotspot analysis of the number of medicinal plant species at different scales. **(A)** is the result at the spatial scale of 50 km; **(B)** is 100 km; **(C)** is 150 km; **(D)** is 200 km.

The results of the hotspot analysis of MPD at different spatial scales are shown in Figure 4. Under the spatial grid of 50 km, there is no distribution of cold point except for a very few spatial grids that pass the 90% confidence level. This indicates that the spatial scale of 50 km only reflects the hot spots status of Inner Mongolia’s MPD (Figure 4A). As the spatial scale became larger, extensive cold spot began to appear in western Inner Mongolia (95% confidence level, 90% confidence level). However, there was no distribution of cold spot that passed the 99% confidence level at either spatial scale. It is noteworthy that although cold spot areas emerged at large spatial scales that were not present in the small-scale analysis, there were also areas that changed from hot spot to insignificant results. For example, at the spatial scale of 200 km, there is no hot spot in Hinggan League, but there are hot spot at other spatial scales, especially at the spatial scales of 50 km and 100 km, it contains hot spot going to reach 99% confidence level.

### 3.4 Exploration of MPD hotspots based on maxent

**FIGURE 5:**
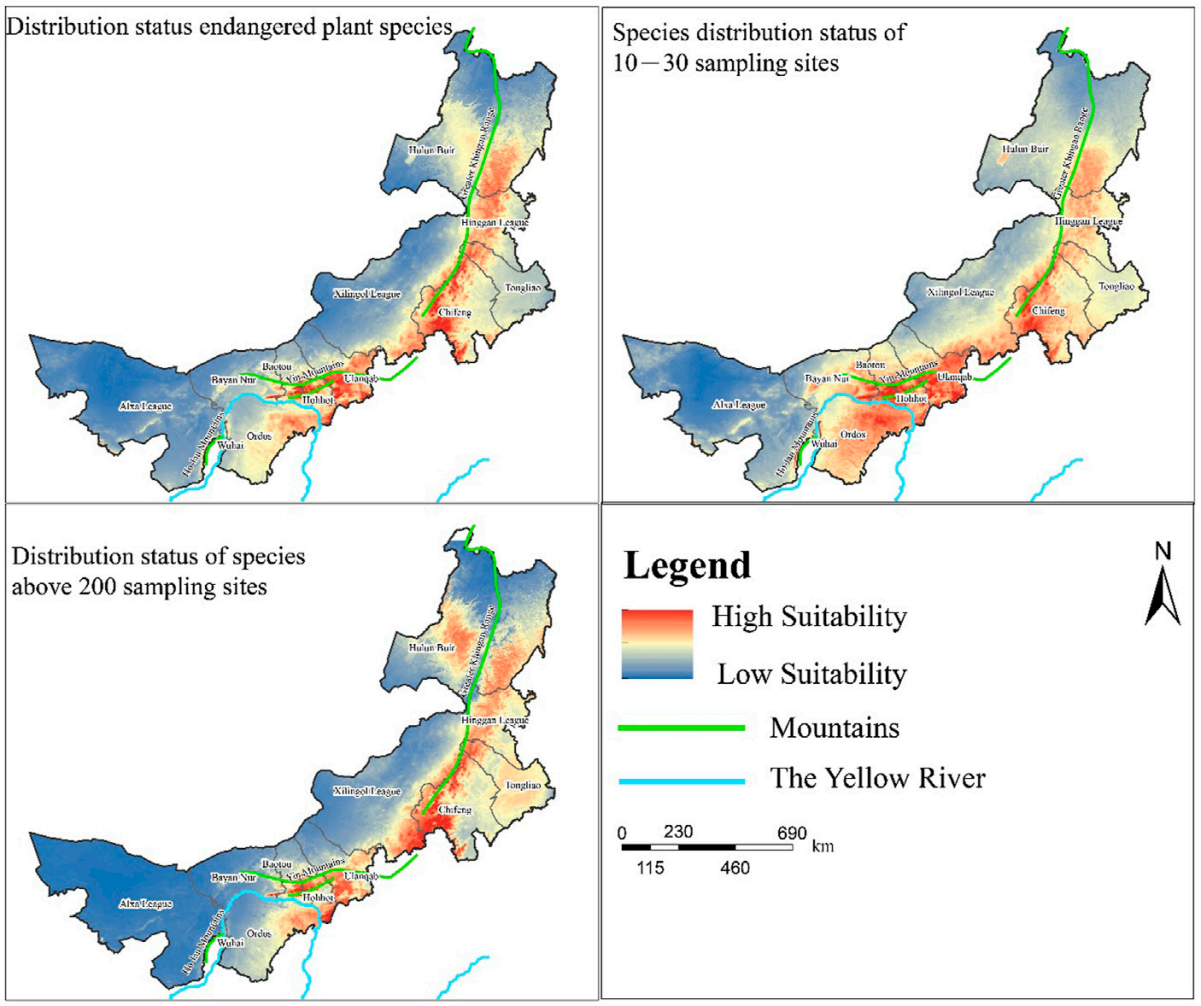
Habitat suitability distribution of medicinal plant diversity in Inner Mongolia.

After considering the overall spatial distribution pattern of the MPD using spatial analyses, we further explored the suitable habitats of the three most representative kinds of medicinal plants using Maxent (Supplementary Table S2). Generally, the suitability habitat of all three kinds of plant species was higher in the vicinity of the Greater Khingan Range and Yinshan Mountains (Figure 5). In the area flowing through the Yellow River, there are fewer species on the west side and more suitable species distribution on the east side. This may be due to the fact that the overlapping areas of the Yinshan Mountains converge with the eastern watershed of the Yellow River, making the local climate more suitable for plant growth, while the western part of Inner Mongolia has an arid climate. Although part of the Yellow River flows through this area, the overall lack of precipitation and other conditions suitable for plant growth reduce the number of plant species. In addition, species with a wide distribution also have areas with highly suitable habitats in the Greater Khingan Range and western Tongliao. This indicates that although the suitability of endangered and rare plants in these two areas is low, some plant ecological suitability remains. Thus, the Yellow River basin and areas along the mountains in Inner Mongolia are well worth the attention of managers and researchers, while the stable low suitability in the western region and the northern border area make them areas that need to be improved in the future.

The first is the trend in the distribution of widely distributed medicinal plant species (Supplementary Figures S1, S2). By 2030, the highly suitable areas for medicinal plants under different SSP are expected to be expanded some extent. Among them, the SSP245 is the development form with the highest suitability for MPD relative to other development pathways. By 2070, the highly suitable areas of each development pathway are expected to be more stable with less variation. Therefore, it can be concluded that the SSP245 has high benefits in long- and short-term development and is effective in maintaining the diversity of widely distributed medicinal plant species.

The second is the trend in the distribution of sparsely distributed medicinal plant species (Supplementary Figures S3, S4). A small expansion of MPD is expected under all four development pathways by 2030, but their subtle trends are not the same. The range of high suitability areas under the SSP245 is the largest among several development pathways, and the other development pathways do not expand areas of high suitability. Under the SSP585, although its suitability area expands in general, the highly suitable areas, such as the suitable areas for medicinal plant diversity in the Yinshan Mountains, have decreased instead. By 2070, the highly suitable areas for MPD contracted under SSP126 and SSP245, and expanded under the SSP370 and SSP585. However, these are accompanied by a migration of the focal areas of medicinal plant diversity, and this trend of migration makes management attention needed in response to climate change impacts.

Finally, there are trends in the distribution of medicinal plant species among endangered species (Supplementary Figures S5, S6). By 2030, the highly suitable areas under SSP126, SSP245, and SSP370 will have expanded slightly. Under the SSP585, the highly suitable areas in the Yinshan Mountains shrink significantly and most of them become moderately suitable areas. It is also noteworthy that the high suitability area under SSP245 has a greater expansion than that under SSP126 and SSP370. Thus, for the conservation of endangered species, the pathway described in SSP245 that follows current socioeconomic and technological trends will have a more positive impact on the development of the area. However, in the long-term development plan (2070), the area of high suitability in the Yinshan Mountains under SSP245 is expected to almost disappear, reflecting the negative impact that the continuation of current socioeconomic policies may have on endangered medicinal plant species.

### 3.5 Analysis of the drivers influencing the richness of MPD

#### 3.5.1 Relationship between ecological data and medicinal plant diversity

**FIGURE 6:**
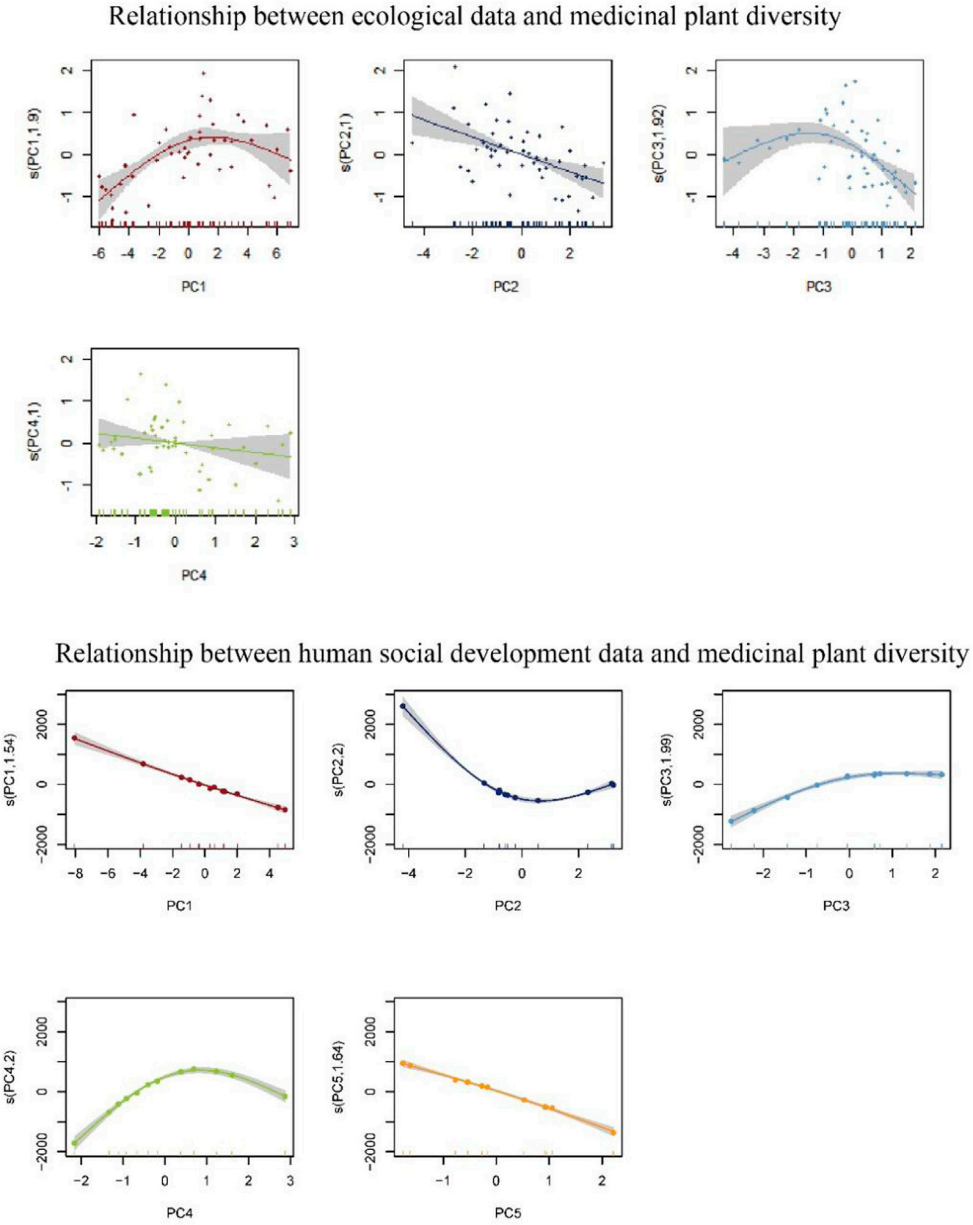
Relationship between each variable and medicinal plants diversity after principal component analysis.

We performed GAM analysis on all four spatial grids. We found that the model fit was the best under a spatial grid of 150 km. After the PC analysis, four PCs were selected with variables explaining 89.86%. The final GAM model fit *R* ^2^ was 0.607, *p* < 0.01, and the model explained deviation reached 65.6%. The trend of the fitted model is shown in Figure 6. The residuals are consistent with the hypothesis, indicating that the model fit is as expected (Supplementary Table S3).

The influence of ecological factors on the trend of MPD changes can be understood by combining the results of the model fitting as shown in Figure 6. And the coefficient reduction equations of each principal component are in Supplementary Material.

In PC1 (*p* < 0.01), the most important influencing factors are the annual mean temperature (bio_1), the max temperature of warmest month (bio_5), the mean temperature of driest quarter (bio_9) and the mean temperature of warmest quarter (bio_10). In PC2 (*p* < 0.01), the important influencing factors include temperature annual range (bio_7), temperature seasonality (bio_4) and isothermality (bio_3). In PC3 (*p* < 0.01), the most important influencing factors can be divided into two categories: elevation and precipitation, where the environmental factors of precipitation are mainly precipitation seasonality (bio_15), precipitation of wettest quarter (bio_16) and precipitation of driest quarter (bio_17). In PC4 (*p* > 0.05), the most important influencing factor was the mean diurnal range (bio_2).

#### 3.5.2 Relationship between human social development data and medicinal plant diversity

The data we collected were different when based on the county and the municipality as statistical units, and we conducted separate GAM analyses. However, the results of the GAM model fitting at the county level are less satisfactory, so we put them in the supplementary material and we mainly analyze the results of the GAM model fitting at the municipal level. From the statistical yearbook and survey data provided by the Inner Mongolia Bureau of Statistics, we compiled data on 25 socioeconomic factors (city-level) that may have influence. After PC analysis, the 25 influential factors were reduced to 5 (cumulative contribution was 89.63%), with *R* ^2^ of 0.996, *p* < 0.01. The trend of the fitted model is shown in Figure 6. The residuals are consistent with the hypothesis, indicating that the model fit is as expected (Supplementary Table S4). According to the coefficient reduction equation, we get the following conclusions:

In PC1 (*p* < 0.01), it is possible that the increase in general public budget medical and health expenditure (GPBMHE) and revenue from pharmaceuticals (RP) will increase the diversity of medicinal plants. In PC2 (*p* < 0.01), the increase in the number of Chinese herbal medicine cultivation enterprises (NCHMCE) and the revenue from proprietary Chinese medicines (RPCM) will increase the MPD. However, the increase of these factors will lead to a certain decrease the MPD after a certain degree of increase. In PC3 (*p* < 0.05), the increase in the cultivated area of Chinese herbal medicine (CACHM) and the total production of Chinese herbs (TPCH) makes MPD decrease. In PC4 (*p* < 0.01), the decrease of city area (CA) and gross domestic product of secondary industry (GDPSI) makes MPD increase. In PC5 (*p* < 0.01), the increase of road mileage (RM) and traditional knowledge of Chinese medicine (CACHM) makes MPD increase.

## 4 Discussion

The loss of diversity due to human activities has changed ecosystem function and stability, and China has effectively worked on diversity in recent decades, but some strategies for improving diversity may conflict with local socioeconomic development (Isbell et al., 2017). Diversity reserves can be extremely effective in protecting diversity (Huang et al., 2020; Mi et al., 2021). In this study, we explored the diversity hotspots in Inner Mongolia using three different methods and perspectives for providing a reference for establishing MPD reserves. Although there is still room for further improvement of the results of this study due to possible data collection, limitations of the analysis method and many other reasons. However, this study is still important for the evaluation of plant diversity in Inner Mongolia and worldwide.

### 4.1 The importance of analysis at different spatial scales

Understanding the determinants of species richness is central to many questions in both pure and applied ecology. Previous studies have shown that differences in spatial scales have different effects for evaluating diversity (Chisholm et al., 2018). However, our knowledge of bioregional-scale diversity is fragmented, creating barriers to systematic conservation planning, and knowledge gaps about diversity at spatial scales deserve more attention (Montalvo-Mancheno et al., 2020). Crawley and Harral proposed that at large spatial scales, species richness depends on the number of species and extinction rates. At smaller spatial scales, species richness depends on the birth, mortality, and dispersal rates of individuals interacting with competitors, mutualists, and natural enemy populations (Crawley and Harral 2001). Thompson et al. (2018) proposed a theoretical understanding that the relationship between diversity and ecosystem functioning varies significantly with the observed spatial scale (Thompson et al., 2018). Therefore, different spatial scales should be carefully selected for analysis when conducting diversity assessments at different spatial scales. In this study, species richness in Inner Mongolia was analyzed for the first time using geographical spatial analyses for evaluating diversity information at different spatial scales. The species richness status in Inner Mongolia at different spatial scales was generally consistent, being richer along the mountain ranges and river basins. However, at finer spatial scales, we observed that different distributions remained; for example, the diversity in the Alxa League had significant cold point aggregation at large spatial scales, but not at small spatial scales. Therefore, a multi-scale spatial analysis study can help researchers and decision-makers to obtain more comprehensive results on regional diversity evaluation and visualize the guidance for diversity reserve planning. Problems due to the division of different spatial scales should also be noted. The uneven spatial grid division at the boundary of Inner Mongolia may cause some analysis errors, which may result in inaccurate spatial analysis of diversity in the boundary zone of the study area. Policymakers and researchers should choose different spatial scales according to the scope of the planned protected areas and the study area.

### 4.2 The importance of using administrative districts as research boundaries life science identifiers

Researchers have verified that the accuracy of expert range maps and global diversity maps can be biased at the junction of administrative boundaries, suggesting that spatial analysis of administrative units is also indispensable when exploring diversity distribution (Abdu-Raheem 2010; Hughes et al., 2021). Ma et al. (2021a) found that using county and district administrative divisions as the unit of analysis had the highest accuracy compared to provincial and municipal administrative divisions (Ma et al., 2021b). Therefore, to provide a better reference for managers’ ecological policies, this study statistically and spatially analyzed the number of species distributions at the county and district levels in Inner Mongolia, and the results were quite different from those obtained after spatial gridding. This suggests that by analyzing diversity between administrative divisions, differences in diversity between urban and suburban areas can be better established and that these differences are results that are not well represented at different spatial scales. Therefore, through the spatial analysis of different administrative units, we can provide clearer guidance on policies for regional management.

### 4.3 Species distribution modeling analysis for MPD research

There is an increased interest in measuring and modeling diversity from space. Biogeographers have a growing interest in species distribution models to simulate species distribution and diversity pattern (Gillespie et al., 2008). Many studies have been conducted to demonstrate that the method used in this study is feasible and reliable. Wu et al. (2018) identified priority areas for grassland endangered plant species in the Sanjiangyuan Nature Reserve based on the Maxent model (Wu et al., 2018). Ma et al., analyzed the hotspot and conservation gaps of bird diversity in the Guangdong Province based on the Maxent model (Ma et al., 2021b). Sohrab et al., identified high-priority conservation areas for avian diversity using the Maxent model (Moradi et al., 2019). Maxent, one of the most widely used models in ecology and related fields, has greatly facilitated the progress of diversity model exploration with its visual interface and easy to interpret statistical results (Phillips et al., 2017). However, the widespread use of Maxent models also requires attention to many issues, such as sample bias, selection of spatial variables, the study scope, and selection of model parameters (Merow et al., 2013). For instance, Field sampling data may be limited by road restrictions, environmental restrictions, and other factors (Elith et al., 2011). The choice of species distribution models still has the potential to improve, and studies have shown that the prediction results of multiple models are more reliable than those of single models (Kaky et al., 2020). For example, the biomod2 model (Thuiller et al., 2009), has been recognized by ecologists with the rapid development of the R (Hao et al., 2019). This model runs up to 10 single models continuously on a presence/absence dataset and merges them into ensemble models and ensemble predictions, and benchmarks for several other evaluation and visualization tools. Therefore, multiple models can better achieve the results of predicting species distribution, which is one of the development directions of diversity model research.

### 4.4 Effect of different factors on MPD

In terms of the natural environment, the annual mean temperature (bio_1), the max temperature of warmest month (bio_5), the mean temperature of driest quarter (bio_9) and the mean temperature of warmest quarter (bio_10) are all related to temperature and negatively correlated with PC1. Therefore, this result shows that temperature has an important influence on MPD. And based on the model of the relationship between PC1 and MPD shown in Figure 6, higher temperature will contribute to the MPD, but not higher temperature is better, there is a certain temperature that has the best contribution to MPD. Temperature annual range (bio_7), temperature seasonality (bio_4) and isothermality (bio_3) are important in PC2, which indicator values obtained from other environmental factors after calculations. In fact, the 19 bioclimatic variables in worldclim are widely used precisely because they are more suitable for biological modeling after certain calculations (Fick and Hijmans, 2017). Therefore, the PC2 can better show the importance of bioclimatic variables on the MPD. As can be seen from Figure 6, PC2 and MPD basically show an approximately linear relationship, with an increase in PC2 leading to a decrease in MPD. Therefore, according to the coefficient reduction equation of PC2, an increase in bio_7 and bio_4 leads to an increase in PC2 and a decrease in MPD, while an increase in bio_4 leads to a decrease in PC2 and an increase in MPD. Elevation and precipitation factors indicate that PC3 mainly reflects the influence of elevation and precipitation on the MPD. According to Figure 6, the trend was similar to the relationship between PC1 and MPD, and there were also these factors that produced the highest MPD within a certain value interval. It is possible that elevation, temperature, and precipitation are correlated, and the complex combination of several factors also has different effects. According to the coefficient reduction equation of PC3, all precipitation factors showed negative correlation with PC3 except for altitude, which was positively correlated with PC3.In PC4, the mean diurnal range (bio_2) is noteworthy that the relationship between mean diurnal range and PC4 is very close and much higher than the influence of other ecological factors. According to the coefficient reduction equation of PC4, the mean diurnal range was positively correlated with PC4, while according to Figure 6, an increase in the mean diurnal range led to a decrease.

From a socio-economic point of view, the increase in general public budget medical and health expenditure (GPBMHE) and revenue from pharmaceuticals (RP) will lower PC1 and increase the diversity of medicinal plants, because these two indicators represent the development of the pharmaceutical industry and the reasonable development and protection of local MPD. The increase in the number of Chinese herbal medicine cultivation enterprises (NCHMCE) and the revenue from proprietary Chinese medicines (RPCM) will lower PC2 and increase the MPD. However, the increase of these factors will lead to a certain decrease the MPD after a certain degree of increase. This indicates that the development of Chinese herbal medicine cultivation and related industries to a certain extent has a positive effect on MPD, but facing over-exploitation or excessive exploitation of farmland will result in a single diversity of medicinal plants. The increase in the cultivated area of Chinese herbal medicine (CACHM) and the total production of Chinese herbs (TPCH) makes PC3 decrease and MPD decrease. These two indicators illustrate the possible inhibitory effect of the development of medicinal plant cultivation on the diversity of medicinal plants. It is necessary for local management to plan the cultivation and cultivation industry of herbal medicine in a reasonable way when planning the conservation of medicinal plant diversity. The decrease of city area (CA) and gross domestic product of secondary industry (GDPSI) makes PC4 and MPD increase. However, when PC4 increases to a certain extent, the MPD will decrease. This shows that a suitable CA and a certain degree of local economic development are suitable for the protection and development of medicinal plant resources, while the MPD in areas with poor economic development or over-developed economies will be reduced. The increase of road mileage (RM) and traditional knowledge of Chinese medicine (CACHM) makes PC5 decrease and the MPD increase. RM represents the local development status while CACHM represents the status of local utilization of medicinal plants. These two factors indicate that PC5 may represent the current situation of local utilization and development of traditional Chinese medicine. With the widespread use and dissemination of medicinal plants, more and more non-native medicinal plants have been disseminated, thereby expanding the local MPD.

### 4.5 Plant diversity and sustainable development

China hosted the Diversity Conference in October 2021, which established the importance of the Fourth National Census of Traditional Chinese Medicine Resources and the rich plant resources laid the foundation for diversity conservation (Ma and Wei, 2021). The Kunming Declaration mentions the need to optimize and establish an effective system for protected areas to promote conservation and restore terrestrial, freshwater, and marine diversity (Zhang et al., 2022). The spatial distribution patterns of plants in Inner Mongolia and their distribution changes under climate change scenarios in this study can be a good response to the main theme of the conference. Simultaneously, the predictive models for future climate selected in this study can provide a research basis for achieving the future development goals of the Conference on Biological Diversity and the long-term vision of plant diversity research.

Conversely, considering the future climate change, sustainable development is gradually becoming the focus of the world’s attention. Three of the Sustainable Development Goals designated by the United Nations Development Programme refer to a relevant development vision (https://www.cn.undp.org/). In Goals 13 and 15, it is evident that the global investment in disaster management under climate change is as high as US$6 billion. Therefore, research on plant diversity under climate change can effectively ensure the stability of terrestrial ecosystems, which is important for terrestrial ecosystems such as forests, wetlands, drylands, and mountains and rivers in Inner Mongolia. Simultaneously, 80% of human food comes from plants, and agriculture and related economies are important means of development. In the GAM model, we explored the effects of different economic factors on MPD. These factors are key influences on controlling climate change, providing universal access to health care, and providing safe and effective medicines as mentioned in Goal 3. Notably, the medicinal plants selected for this study imply that the ideas of this study are important for the development of the traditional pharmaceutical industry on a larger scale as an approach that can be extended to a global scale.

## 5 Conclusion

This study adopts a multidisciplinary approach to investigate the spatial distribution patterns of medicinal plant diversity in Inner Mongolia from different dimensions and to demonstrate the possible trends of changes in medicinal plant diversity under future climate. At the same time, environmental factors and socio-economic development were organically combined and non-linear relationships were established. The western region of Inner Mongolia is the main cold spot of MPD, and medicinal plant resources are relatively scarce in this region; the central and northeastern regions of Inner Mongolia are the main hot spots of MPD with relatively abundant medicinal plant resources. Under the future climate change, the areas with high habitat suitability for medicinal plant diversity are still mainly around the Yellow River, Yin Mountains and Greater Khingan Range.It is worth noting that the development path of SSP245 is still a reference path for the maintenance of medicinal plant resource diversity in both the long-term and short-term development. The nonlinear relationships of the drivers of medicinal plant diversity at different spatial scales constructed by the generalized summation model demonstrate that temperature, precipitation, and socioeconomic development conditions do have a complex effect on medicinal plant diversity. This study, which constructs the relationship between ecological and socioeconomic factors on the driven influence of medicinal plants through a multidimensional approach, provides a reference for in-depth research exploring the influence of complex factors on natural resources.

## Data Availability

The original contributions presented in the study are included in the article/Supplementary Material, further inquiries can be directed to the corresponding authors.
