# Supplementary material for: Complex ecological and socioeconomic impacts on medicinal plant diversity
Source: Front Pharmacol. 2022 Oct 19;13:979890. doi: 10.3389/fphar.2022.979890 (PMC9627218; doi:10.3389/fphar.2022.979890)
Supplement: Supplementary file 1 [file DataSheet1.docx]

Supplementary Material

# Supplementary Figure


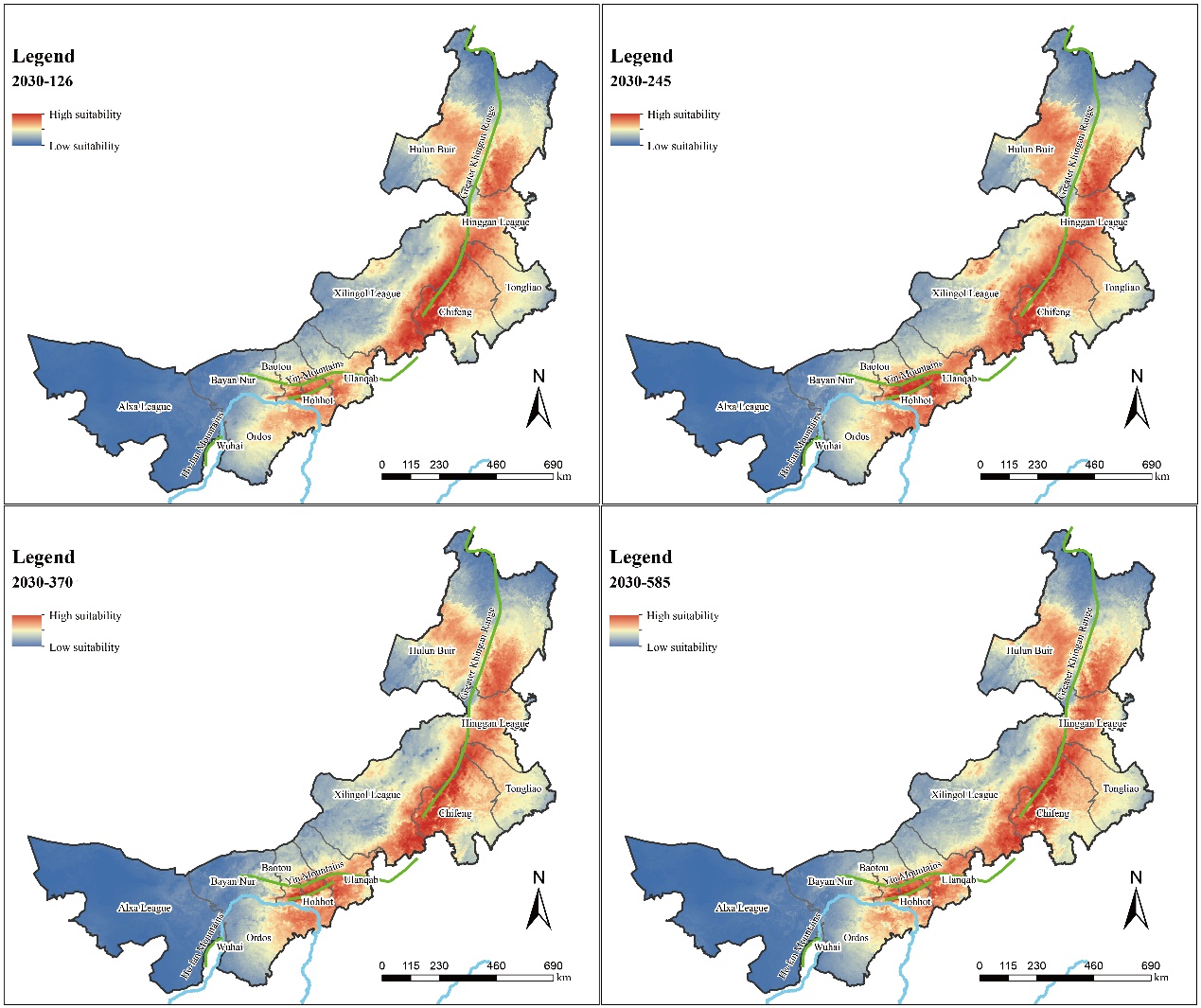


**Supplementary Figure 1.** Habitat suitability of MPD of widely distributed species under four pathways in 2030.


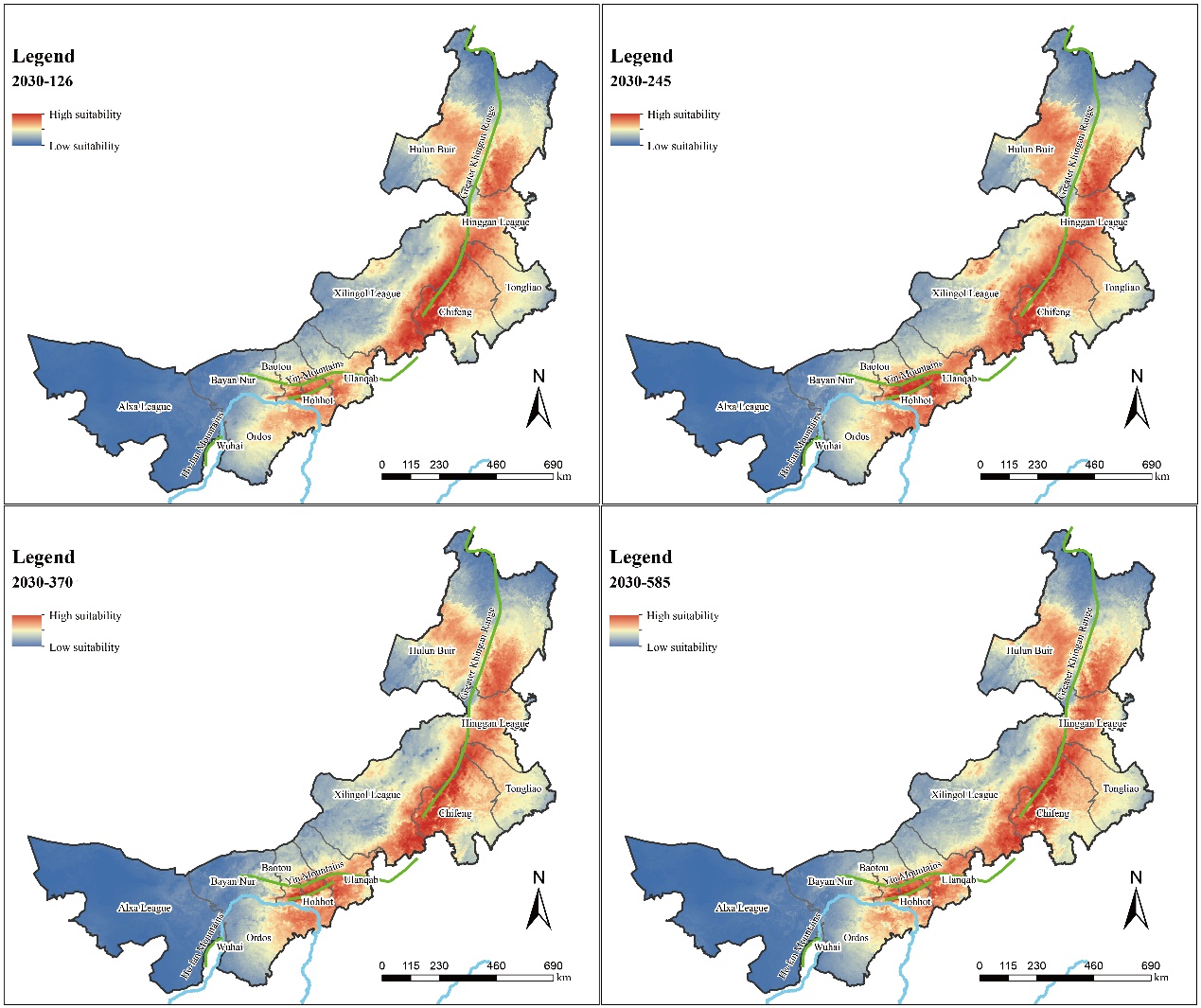


**Supplementary** **Figure 2.** Habitat suitability of MPD of widely distributed species under four pathways in 2070.


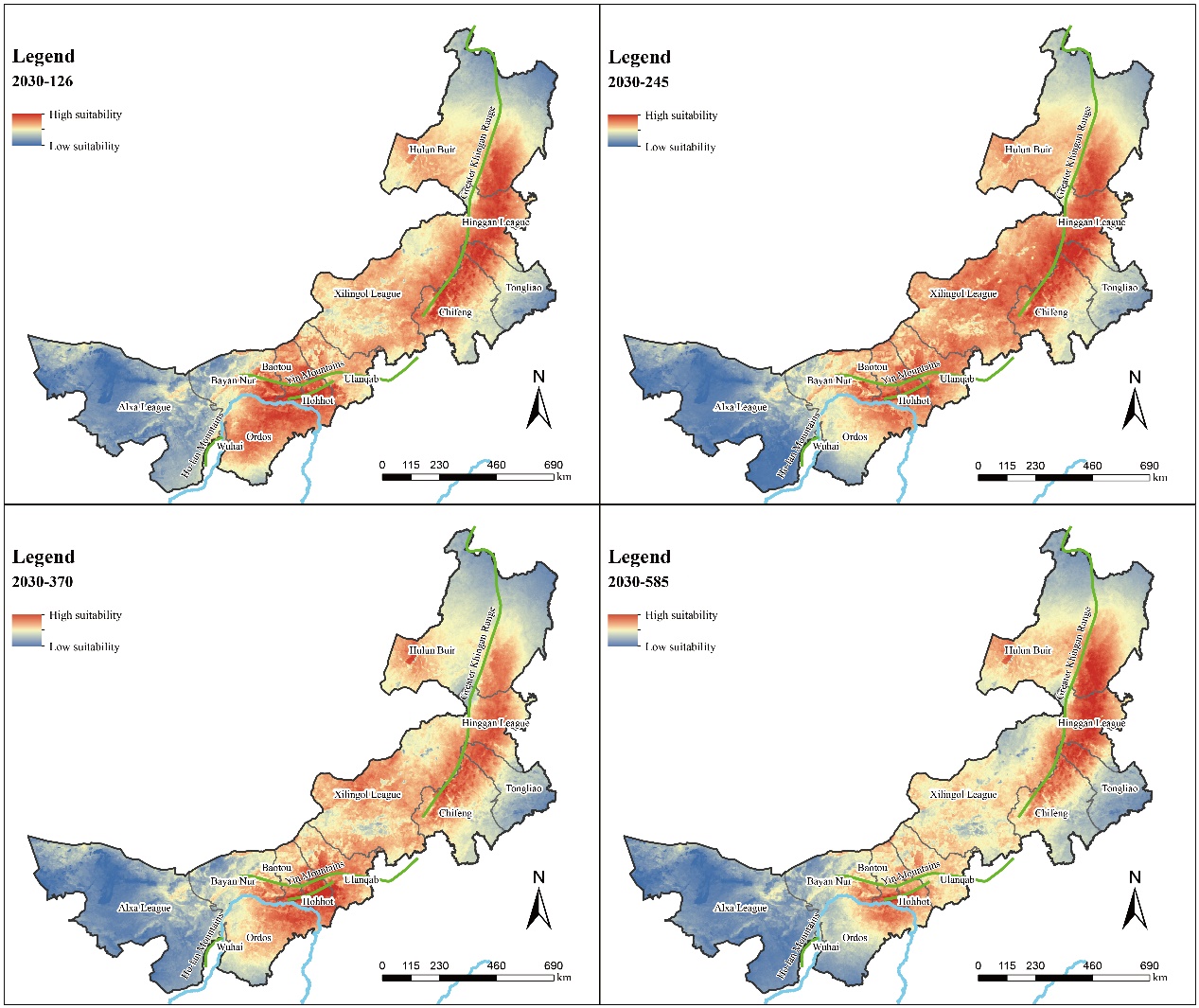


**Supplementary Figure 3.** Habitat suitability of MPD of sparsely distributed species under four pathways in 2030.


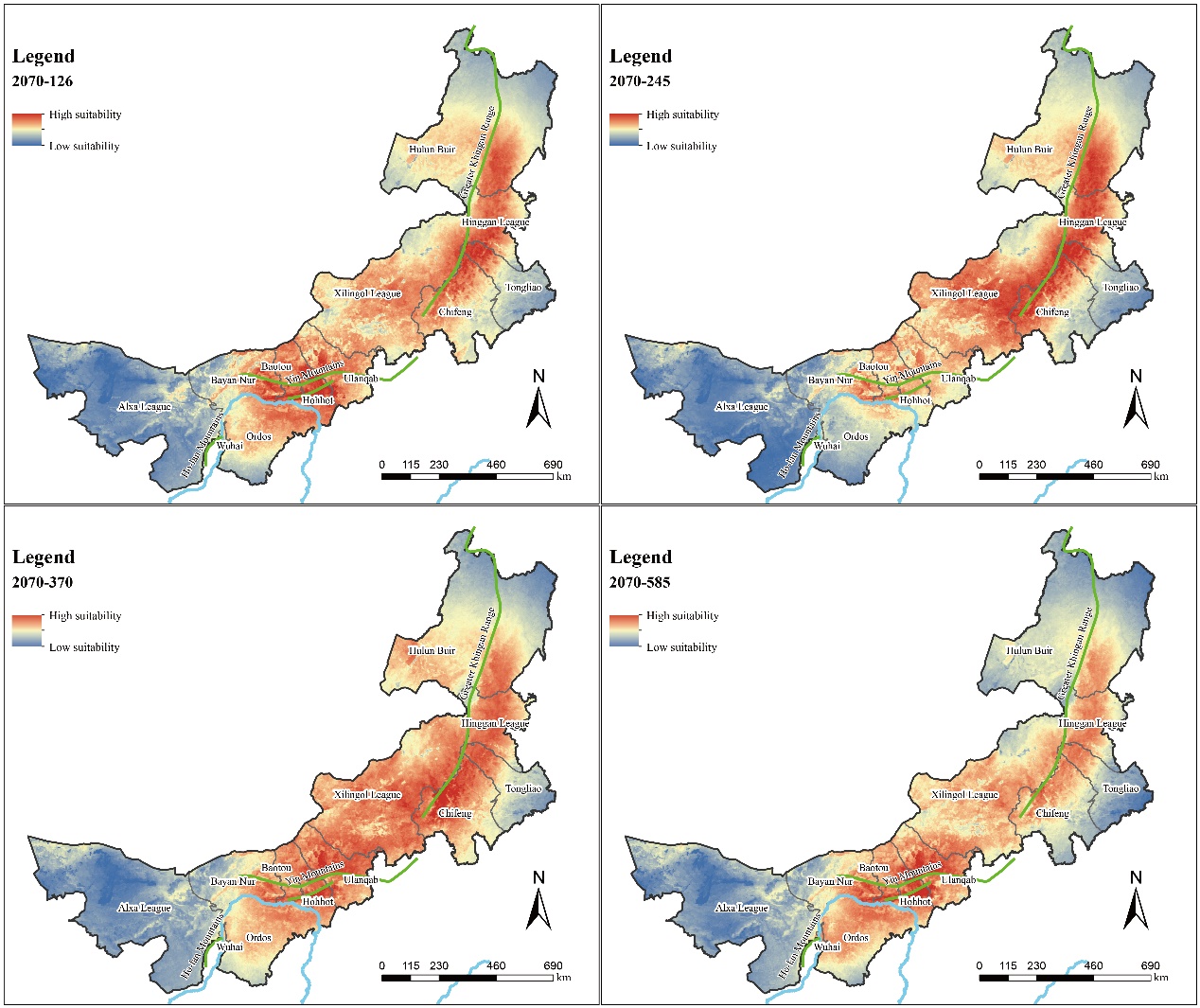


**Supplementary Figure 4.** Habitat suitability of MPD of sparsely distributed species under four pathways in 2070.


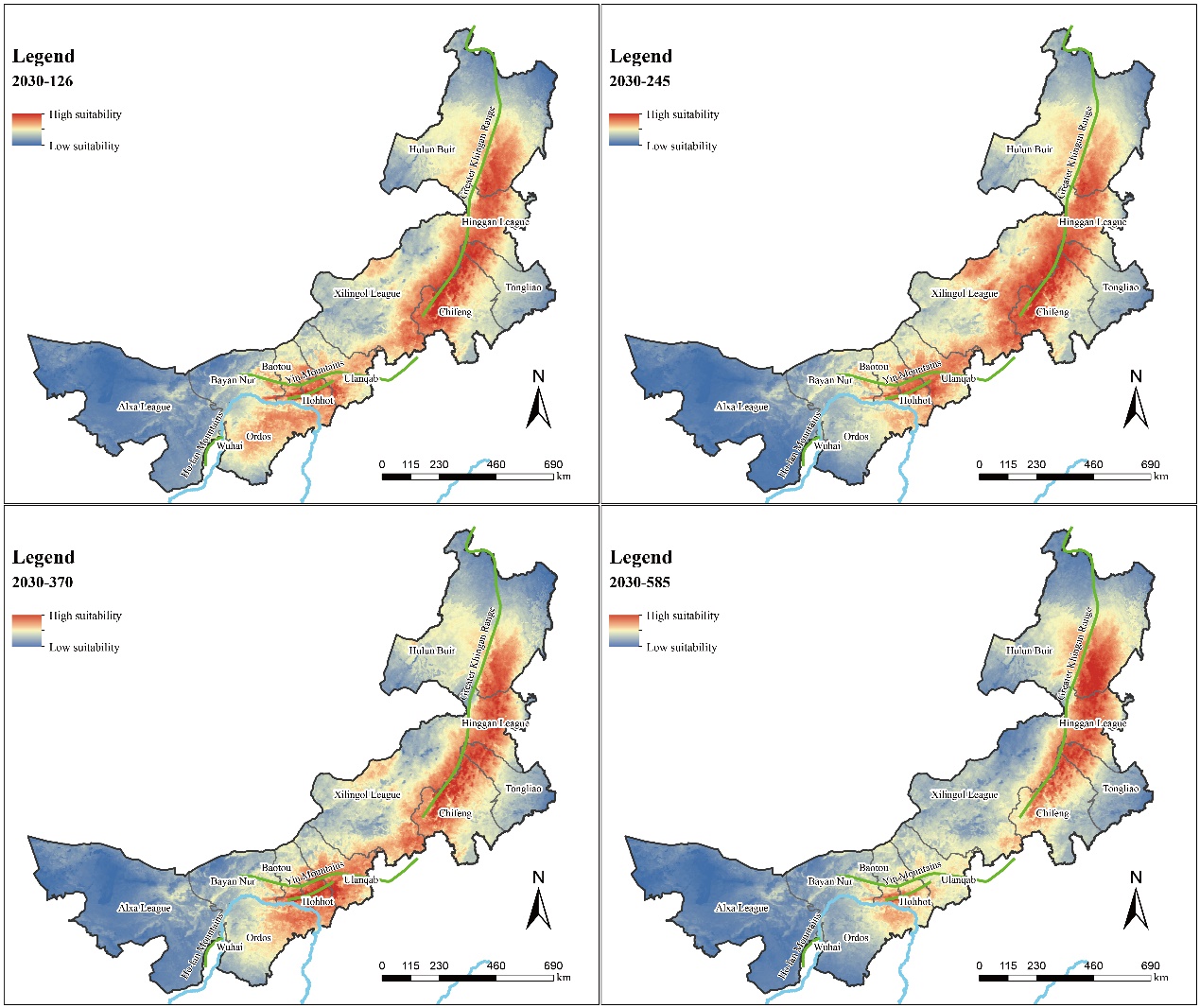


**Supplementary Figure 5.** Habitat suitability of MPD of endangered species under four pathways in 2030


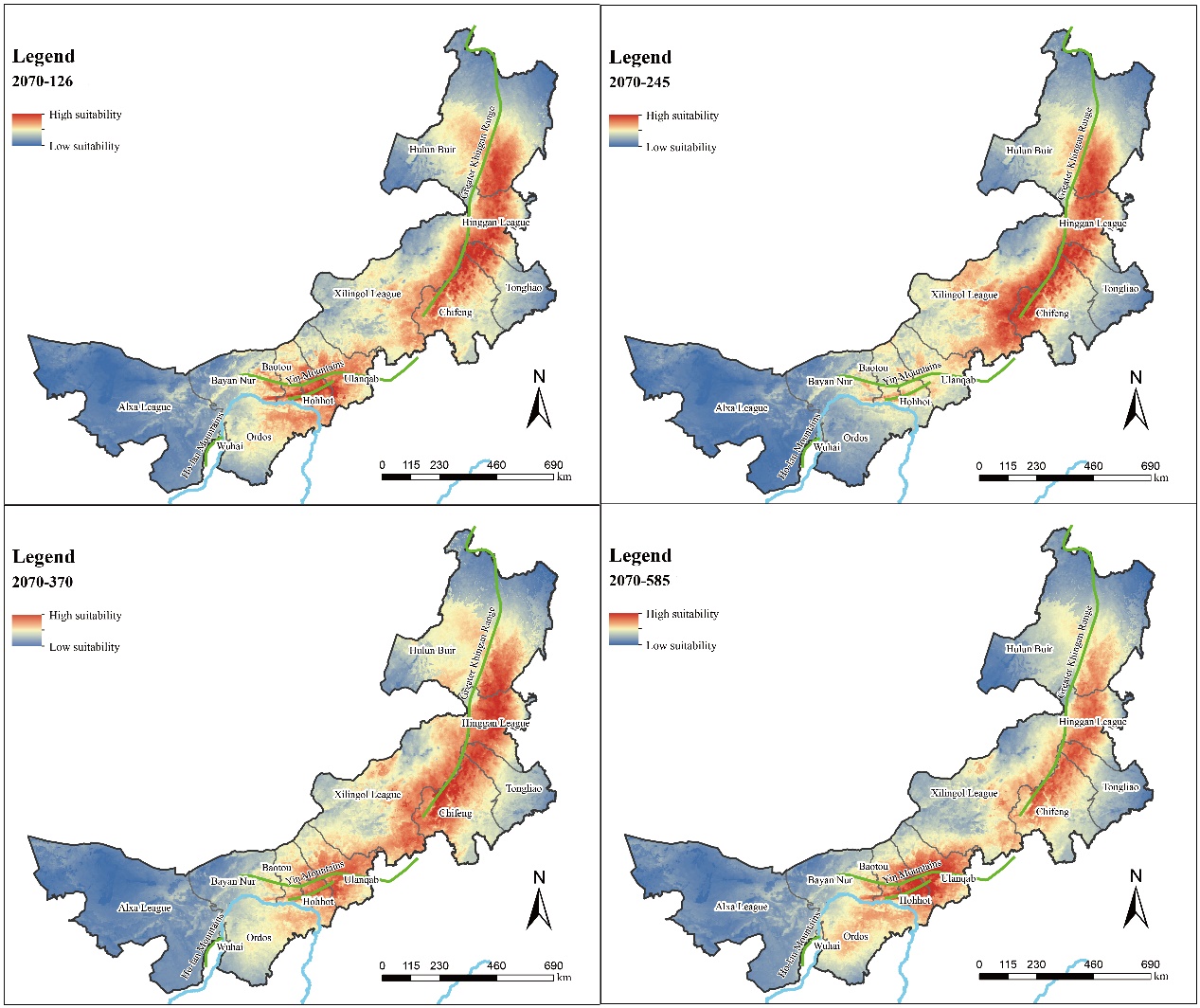


**Supplementary** **Figure 6.** Habitat suitability of MPD of endangered species under four pathways in 2070.

# Supplementary Tables

**Supplementary Table 1**. Model fitting results

| Data Type | Abbreviations | Data source |
| --- | --- | --- |
| City | City | Inner Mongolia Statistical Yearbook |
| General public budget medical and health expenditure | GPBMHE |  |
| Gross domestic product of secondary industry | GDPSI |  |
| Gross domestic product of tertiary industry | GDPTI |  |
| Gross domestic product of primary industry | GDPPI |  |
| Gross domestic product | GDP |  |
| Total arable land area | TLA |  |
| Number of health institutions | NHI |  |
| Number of health facility personnel | NHFP |  |
| City Area | CA |  |
| Road mileage | RM |  |
| Number of varieties traded in Chinese herbal market | NVTCHM | Fourth National Census of Traditional Chinese Medicine Resources |
| Traditional Knowledge of Chinese Medicine | TKCM |  |
| Total production of Chinese herbs | TPCH |  |
| Cultivated area of Chinese herbal medicine. | CACHM |  |
| Number of herbal cultivation species | NHCS |  |
| Number of Chinese herbal medicine cultivation enterprises | NCHMCE | Inner Mongolia Bureau of Statistics |
| Number of Chinese medicine production and processing enterprises | NCMPPE |  |
| Number of Chinese medicine wholesale enterprises | NCMWE |  |
| Number of Chinese medicine retail enterprises | NCMRE |  |
| Number of Chinese herbal medicine storage enterprises | NCHMSE |  |
| Population | Population | The seventh National Census |
| Revenue from Pharmaceuticals | RP | China Academy of Chinese Medical Sciences |
| Revenue from Western medicine | RWM |  |
| Revenue from Chinese herbal medicine | RCHM |  |
| Revenue from proprietary Chinese medicines | RPCM |  |

**Supplementary Table 2.** Autocorrelation of the MPD at different scales

| Different scales | Moran's I | Z | *P* | Spatial Correlation |
| --- | --- | --- | --- | --- |
| Administrative district | 0.341992 | 2.439759 | 0.01 | Positive |
| 50 km | 0.460951 | 15.192380 | 0.00 | Positive |
| 100 km | 0.559819 | 9.888233 | 0.00 | Positive |
| 150 km | 0.583574 | 7.286754 | 0.00 | Positive |
| 200 km | 0.544882 | 5.377031 | 0.00 | Positive |

**Supplementary Table 3.** List of plants from three sources in Maxent analysis

| Species | Source | Species | Source | Species | Source | Species | Source |
| --- | --- | --- | --- | --- | --- | --- | --- |
| *Acalypha australis* | 10–30 points | *Diarthron linifolium* | 10–30 points | *Pennisetum centrasiaticum* | 10–30 points | *Vicia cracca* | 10–30 points |
| *Acer ginnala* | 10–30 points | *Digitaria ischaemum* | 10–30 points | *Petunia hybrida* | 10–30 points | *Vicia gigantea* | 10–30 points |
| *Acer mono* | 10–30 points | *Dioscorea nipponica* | 10–30 points | *Pharbitis purpurea* | 10–30 points | *Vicia multicaulis* | 10–30 points |
| *Acer negundo* | 10–30 points | *Draba nemorosa* | 10–30 points | *Phaseolus vulgaris* | 10–30 points | *Vicia pseudorobus* | 10–30 points |
| *Acer truncatum* | 10–30 points | *Dracocephalum argunense* | 10–30 points | *Phlomis dentosa* | 10–30 points | *Viola acuminata* | 10–30 points |
| *Achillea acuminata* | 10–30 points | *Echinochloa crusgalli* | 10–30 points | *Physochlaina physaloides* | 10–30 points | *Viola dissecta* | 10–30 points |
| *Achillea millefolium* | 10–30 points | *Echinochloa phyllopogon* | 10–30 points | *Picea wilsonii* | 10–30 points | *Viola prionantha* | 10–30 points |
| *Achnatherum inebrians* | 10–30 points | *Echinops latifolius* | 10–30 points | *Pimpinella thellungiana* | 10–30 points | *Weigela florida* | 10–30 points |
| *Aconitum vilmorinianum* | 10–30 points | *Echinops przewalskii* | 10–30 points | *Plantago major* | 10–30 points | *Xanthium mongolicum* | 10–30 points |
| *Acroptilon repens* | 10–30 points | *Elsholtzia ciliata* | 10–30 points | *Plantago minuta* | 10–30 points | *Xanthoceras sorbifolium* | 10–30 points |
| *Adenophora polyantha* | 10–30 points | *Elsholtzia densa* | 10–30 points | *Poa pratensis* | 10–30 points | *Youngia tenuifolia* | 10–30 points |
| *Adina rubella* | 10–30 points | *Elymus dahuricus* | 10–30 points | *Poa sphondylodes* | 10–30 points | *Zinnia elegans* | 10–30 points |
| *Agastache rugosa* | 10–30 points | *Elymus nutans* | 10–30 points | *Polygala sibirica* | 10–30 points | *Ziziphus jujuba* | 10–30 points |
| *Agropyron desertorum* | 10–30 points | *Enneapogon borealis* | 10–30 points | *Polygonum alopecuroides* | 10–30 points | *Zygophyllum mucronatum* | 10–30 points |
| *Agropyron mongolicum* | 10–30 points | *Ephedra equisetina* | 10–30 points | *Polygonum amphibium* | 10–30 points | *Aconitum kusnezoffii* | List of Key Protected Wild Plants in Inner Mongolia Autonomous Region |
| *Allium bidentatum* | 10–30 points | *Ephedra przewalskii* | 10–30 points | *Polygonum sieboldii* | 10–30 points | *Adenophora gmelinii* | List of Key Protected Wild Plants in Inner Mongolia Autonomous Region |
| *Allium chrysanthum* | 10–30 points | *Equisetum hyemale* | 10–30 points | *Polygonum taquetii* | 10–30 points | *Adenophora stenanthina* | List of Key Protected Wild Plants in Inner Mongolia Autonomous Region |
| *Allium fistulosum* | 10–30 points | *Equisetum palustre* | 10–30 points | *Populus euphratica* | 10–30 points | *Adenophora tetraphylla* | List of Key Protected Wild Plants in Inner Mongolia Autonomous Region |
| *Allium ledebourianum* | 10–30 points | *Equisetum ramosissimum* | 10–30 points | *Potaninia mongolica* | 10–30 points | *Agropyron mongolicum* | List of Key Protected Wild Plants in Inner Mongolia Autonomous Region |
| *Allium polyrhizum* | 10–30 points | *Equisetum sylvaticum* | 10–30 points | *Potentilla bifurca* | 10–30 points | *Alisma orientale* | List of Key Protected Wild Plants in Inner Mongolia Autonomous Region |
| *Allium tuberosum* | 10–30 points | *Eragrostis minor* | 10–30 points | *Potentilla conferta* | 10–30 points | *Allium mogolicum* | List of Key Protected Wild Plants in Inner Mongolia Autonomous Region |
| *Althaea rosea* | 10–30 points | *Eragrostis pilosa* | 10–30 points | *Potentilla discolor* | 10–30 points | *Ammopiptanthus  mongolicus* | List of Key Protected Wild Plants in Inner Mongolia Autonomous Region |
| *Amaranthus blitoides* | 10–30 points | *Erigeron acer* | 10–30 points | *Potentilla glabra* | 10–30 points | *Anemarrhena asphodeloides* | List of Key Protected Wild Plants in Inner Mongolia Autonomous Region |
| *Ammopiptanthus mongolicus* | 10–30 points | *Eriochloa villosa* | 10–30 points | *Potentilla longifolia* | 10–30 points | *Anemone silvestris* | List of Key Protected Wild Plants in Inner Mongolia Autonomous Region |
| *Amorpha fruticosa* | 10–30 points | *Eritrichium mandshuricum* | 10–30 points | *Potentilla multicaulis* | 10–30 points | *Apocynum venetum* | List of Key Protected Wild Plants in Inner Mongolia Autonomous Region |
| *Amygdalus pedunculata* | 10–30 points | *Erodium cicutarium* | 10–30 points | *Potentilla multifida* | 10–30 points | *Astragalus complanatus* | List of Key Protected Wild Plants in Inner Mongolia Autonomous Region |
| *Androsace filiformis* | 10–30 points | *Eruca sativa* | 10–30 points | *Potentilla parvifolia* | 10–30 points | *Astragalus mongholicus* | List of Key Protected Wild Plants in Inner Mongolia Autonomous Region |
| *Androsace incana* | 10–30 points | *Erysimum bungei* | 10–30 points | *Potentilla sericea* | 10–30 points | *Atractylodes lancea* | List of Key Protected Wild Plants in Inner Mongolia Autonomous Region |
| *Androsace mariae* | 10–30 points | *Erysimum cheiranthoides* | 10–30 points | *Primula farinosa* | 10–30 points | *Atraphaxis bracteata* | List of Key Protected Wild Plants in Inner Mongolia Autonomous Region |
| *Androsace maxima* | 10–30 points | *Erysimum flavum* | 10–30 points | *Prunus cerasifera* | 10–30 points | *Berberis caroli* | List of Key Protected Wild Plants in Inner Mongolia Autonomous Region |
| *Androsace umbellata* | 10–30 points | *Euonymus bungeanus* | 10–30 points | *Psammochloa villosa* | 10–30 points | *Bupleurum scorzonerifolium* | List of Key Protected Wild Plants in Inner Mongolia Autonomous Region |
| *Anemone narcissiflora* | 10–30 points | *Euonymus maackii* | 10–30 points | *Pteridium aquilinum* | 10–30 points | *Bupleurum sibiricum* | List of Key Protected Wild Plants in Inner Mongolia Autonomous Region |
| *Anemone silvestris* | 10–30 points | *Euphrasia pectinata* | 10–30 points | *Puccinellia distans* | 10–30 points | *Calligonum mongolicum* | List of Key Protected Wild Plants in Inner Mongolia Autonomous Region |
| *Anthriscus sylvestris* | 10–30 points | *Fagopyrum esculentum* | 10–30 points | *Pugionium cornutum* | 10–30 points | *Campanula punctata* | List of Key Protected Wild Plants in Inner Mongolia Autonomous Region |
| *Apocynum venetum* | 10–30 points | *Filipendula palmata* | 10–30 points | *Pyrola rotundifolia* | 10–30 points | *Caragana roborovskyi* | List of Key Protected Wild Plants in Inner Mongolia Autonomous Region |
| *Arctium lappa* | 10–30 points | *Flueggea suffruticosa* | 10–30 points | *Quercus wutaishanica* | 10–30 points | *Caryopteris mongholica* | List of Key Protected Wild Plants in Inner Mongolia Autonomous Region |
| *Arenaria capillaris* | 10–30 points | *Foeniculum vulgare* | 10–30 points | *Rabdosia japonica* | 10–30 points | *Cimicifuga dahurica* | List of Key Protected Wild Plants in Inner Mongolia Autonomous Region |
| *Arenaria juncea* | 10–30 points | *Forsythia suspensa* | 10–30 points | *Ranunculus repens* | 10–30 points | *Clematis fruticosa* | List of Key Protected Wild Plants in Inner Mongolia Autonomous Region |
| *Armeniaca sibirica* | 10–30 points | *Fraxinus chinensis* | 10–30 points | *Ranunculus sceleratus* | 10–30 points | *Cnidium monnier* | List of Key Protected Wild Plants in Inner Mongolia Autonomous Region |
| *Armeniaca vulgaris* | 10–30 points | *Gagea pauciflora* | 10–30 points | *Rhamnus erythroxylon* | 10–30 points | *Cynanchum komarovii* | List of Key Protected Wild Plants in Inner Mongolia Autonomous Region |
| *Arnebia guttata* | 10–30 points | *Gaillardia aristata* | 10–30 points | *Rhamnus ussuriensis* | 10–30 points | *Cynanchum paniculatum* | List of Key Protected Wild Plants in Inner Mongolia Autonomous Region |
| *Artemisia anethoides* | 10–30 points | *Galeopsis bifida* | 10–30 points | *Rheum franzenbachii* | 10–30 points | *Cynomorium songaricum* | List of Key Protected Wild Plants in Inner Mongolia Autonomous Region |
| *Artemisia argyi* | 10–30 points | *Galinsoga parviflora* | 10–30 points | *Rheum undulatum* | 10–30 points | *Dictamnus dasycarpus* | List of Key Protected Wild Plants in Inner Mongolia Autonomous Region |
| *Artemisia blepharolepis* | 10–30 points | *Galium aparine* | 10–30 points | *Rhododendron micranthum* | 10–30 points | *Elsholtzia ciliata* | List of Key Protected Wild Plants in Inner Mongolia Autonomous Region |
| *Artemisia carvifolia* | 10–30 points | *Gentiana scabra* | 10–30 points | *Rhus typhina* | 10–30 points | *Ephedra intermedia* | List of Key Protected Wild Plants in Inner Mongolia Autonomous Region |
| *Artemisia desertorum* | 10–30 points | *Gentiana triflora* | 10–30 points | *Ribes diacanthum* | 10–30 points | *Ephedra equisetina* | List of Key Protected Wild Plants in Inner Mongolia Autonomous Region |
| *Artemisia dracunculus* | 10–30 points | *Gentianella acuta* | 10–30 points | *Ribes pulchellum* | 10–30 points | *Ephrdra sinica* | List of Key Protected Wild Plants in Inner Mongolia Autonomous Region |
| *Artemisia eriopoda* | 10–30 points | *Geranium dahuricum* | 10–30 points | *Robinia pseudoacacia* | 10–30 points | *Equisetum arvense* | List of Key Protected Wild Plants in Inner Mongolia Autonomous Region |
| *Artemisia gmelinii* | 10–30 points | *Geranium maximowiczii* | 10–30 points | *Rosa acicularis* | 10–30 points | *Gentiana dohurica* | List of Key Protected Wild Plants in Inner Mongolia Autonomous Region |
| *Artemisia ordosica* | 10–30 points | *Geranium pratense* | 10–30 points | *Rosa xanthina* | 10–30 points | *Glycine soja* | List of Key Protected Wild Plants in Inner Mongolia Autonomous Region |
| *Artemisia oxycephala* | 10–30 points | *Geranium wlassowianum* | 10–30 points | *Rubus arcticus* | 10–30 points | *Haloxylon  ammodendron* | List of Key Protected Wild Plants in Inner Mongolia Autonomous Region |
| *Artemisia palustris* | 10–30 points | *Glycine max* | 10–30 points | *Rubus sachalinensis* | 10–30 points | *Haplophyllum tragacanthoides* | List of Key Protected Wild Plants in Inner Mongolia Autonomous Region |
| *Aruncus sylvester* | 10–30 points | *Gueldenstaedtia stenophylla* | 10–30 points | *Rubus taiwanianus* | 10–30 points | *Hemerocallis minor* | List of Key Protected Wild Plants in Inner Mongolia Autonomous Region |
| *Asparagus gobicus* | 10–30 points | *Gueldenstaedtia verna* | 10–30 points | *Rudbeckia hirta* | 10–30 points | *Leonurus sibiricus* | List of Key Protected Wild Plants in Inner Mongolia Autonomous Region |
| *Asparagus schoberioides* | 10–30 points | *Gymnadenia conopsea* | 10–30 points | *Rumex acetosa* | 10–30 points | *Lilium dauricum* | List of Key Protected Wild Plants in Inner Mongolia Autonomous Region |
| *Asparagus trichophyllus* | 10–30 points | *Gypsophila davurica* | 10–30 points | *Rumex acetosella* | 10–30 points | *Lilium pumilum* | List of Key Protected Wild Plants in Inner Mongolia Autonomous Region |
| *Aster alpinus* | 10–30 points | *Gypsophila licentiana* | 10–30 points | *Rumex crispus* | 10–30 points | *Limonium bicolor* | List of Key Protected Wild Plants in Inner Mongolia Autonomous Region |
| *Asterothamnus centraliasiaticus* | 10–30 points | *Halogeton arachnoideus* | 10–30 points | *Rumex gmelinii* | 10–30 points | *Medicago falcata* | List of Key Protected Wild Plants in Inner Mongolia Autonomous Region |
| *Astragalus discolor* | 10–30 points | *Haplophyllum tragacanthoides* | 10–30 points | *Rumex thyrsiflorus* | 10–30 points | *Mentha haplocalyx* | List of Key Protected Wild Plants in Inner Mongolia Autonomous Region |
| *Astragalus galactites* | 10–30 points | *Hedysarum brachypterum* | 10–30 points | *Rumex trisetifer* | 10–30 points | *Orobanche pycnostachya* | List of Key Protected Wild Plants in Inner Mongolia Autonomous Region |
| *Atraphaxis bracteata* | 10–30 points | *Hedysarum fruticosum* | 10–30 points | *Sagittaria trifolia* | 10–30 points | *Paeonia lactiflora* | List of Key Protected Wild Plants in Inner Mongolia Autonomous Region |
| *Atriplex centralasiatica* | 10–30 points | *Hedysarum gmelinii* | 10–30 points | *Salix alba* | 10–30 points | *Paeonia lactiflora* | List of Key Protected Wild Plants in Inner Mongolia Autonomous Region |
| *Atriplex sibirica* | 10–30 points | *Hedysarum polybotrys* | 10–30 points | *Salix babylonica* | 10–30 points | *Panzerina lanata* | List of Key Protected Wild Plants in Inner Mongolia Autonomous Region |
| *Axyris hybrida* | 10–30 points | *Heleocharis intersita* | 10–30 points | *Salix cheilophila* | 10–30 points | *Platycodon grandiflorus* | List of Key Protected Wild Plants in Inner Mongolia Autonomous Region |
| *Beckmannia syzigachne* | 10–30 points | *Helianthus annuus* | 10–30 points | *Salix gordejevii* | 10–30 points | *Polygala sibirica* | List of Key Protected Wild Plants in Inner Mongolia Autonomous Region |
| *Berberis caroli* | 10–30 points | *Helianthus tuberosus* | 10–30 points | *Salix matsudana* | 10–30 points | *Polygala tenuifolia* | List of Key Protected Wild Plants in Inner Mongolia Autonomous Region |
| *Bidens pilosa* | 10–30 points | *Hemerocallis fulva* | 10–30 points | *Salix psammophila* | 10–30 points | *Polygonatum odoratum* | List of Key Protected Wild Plants in Inner Mongolia Autonomous Region |
| *Bothriospermum kusnezowii* | 10–30 points | *Heracleum moellendorffii* | 10–30 points | *Salsola laricifolia* | 10–30 points | *Polygonatum sibiricum* | List of Key Protected Wild Plants in Inner Mongolia Autonomous Region |
| *Brassica juncea* | 10–30 points | *Hieracium umbellatum* | 10–30 points | *Salvia japonica* | 10–30 points | *Potaninia  mongolica* | List of Key Protected Wild Plants in Inner Mongolia Autonomous Region |
| *Bromus inermis* | 10–30 points | *Hierochloe glabra* | 10–30 points | *Sambucus williamsii* | 10–30 points | *Potentilla glabra* | List of Key Protected Wild Plants in Inner Mongolia Autonomous Region |
| *Bupleurum longiradiatum* | 10–30 points | *Hippuris vulgaris* | 10–30 points | *Saussurea alata* | 10–30 points | *Prunus mongolica* | List of Key Protected Wild Plants in Inner Mongolia Autonomous Region |
| *Butomus umbellatus* | 10–30 points | *Humulus scandens* | 10–30 points | *Saussurea pulchella* | 10–30 points | *Prunus  mongolica* | List of Key Protected Wild Plants in Inner Mongolia Autonomous Region |
| *Calamagrostis epigeios* | 10–30 points | *Hylotelephium pallescens* | 10–30 points | *Scirpus planiculmis* | 10–30 points | *Ptentilla parvifolia* | List of Key Protected Wild Plants in Inner Mongolia Autonomous Region |
| *Calamagrostis pseudophragmites* | 10–30 points | *Hylotelephium purpureum* | 10–30 points | *Scirpus validus* | 10–30 points | *Pugionium cornutum* | List of Key Protected Wild Plants in Inner Mongolia Autonomous Region |
| *Calligonum mongolicum* | 10–30 points | *Hyoscyamus bohemicus* | 10–30 points | *Scorzonera albicaulis* | 10–30 points | *Pulsatilla chinensis* | List of Key Protected Wild Plants in Inner Mongolia Autonomous Region |
| *Callistephus chinensis* | 10–30 points | *Hypericum ascyron* | 10–30 points | *Scorzonera divaricata* | 10–30 points | *Rheum franzenbachii* | List of Key Protected Wild Plants in Inner Mongolia Autonomous Region |
| *Caltha palustris* | 10–30 points | *Hypericum attenuatum* | 10–30 points | *Scorzonera mongolica* | 10–30 points | *Rhododendron dauricum* | List of Key Protected Wild Plants in Inner Mongolia Autonomous Region |
| *Calvatia gigantea* | 10–30 points | *Hypericum longistylum* | 10–30 points | *Scorzonera pseudodivaricata* | 10–30 points | *Rosa rugosa* | List of Key Protected Wild Plants in Inner Mongolia Autonomous Region |
| *Calystegia pellita* | 10–30 points | *Hypochaeris ciliata* | 10–30 points | *Scutellaria regeliana* | 10–30 points | *Sedum aizoon* | List of Key Protected Wild Plants in Inner Mongolia Autonomous Region |
| *Calystegia sepium* | 10–30 points | *Impatiens noli-tangere* | 10–30 points | *Sedum aizoon* | 10–30 points | *Sophora alopecuroides* | List of Key Protected Wild Plants in Inner Mongolia Autonomous Region |
| *Campanula puncatata* | 10–30 points | *Imperata cylindrica* | 10–30 points | *Selaginella sinensis* | 10–30 points | *Sophora flavescens* | List of Key Protected Wild Plants in Inner Mongolia Autonomous Region |
| *Capsicum annuum* | 10–30 points | *Iris bungei* | 10–30 points | *Selaginella tamariscina* | 10–30 points | *Spiranthes   sinensis* | List of Key Protected Wild Plants in Inner Mongolia Autonomous Region |
| *Caragana brachypoda* | 10–30 points | *Iris ruthenica* | 10–30 points | *Senecio argunensis* | 10–30 points | *Stellaria dichotoma Var. linearis* | List of Key Protected Wild Plants in Inner Mongolia Autonomous Region |
| *Caragana korshinskii* | 10–30 points | *Iris sanguinea* | 10–30 points | *Senecio cannabifolius* | 10–30 points | *Takeikadczukia lomonossowii* | List of Key Protected Wild Plants in Inner Mongolia Autonomous Region |
| *Caragana roborovskyi* | 10–30 points | *Iris tectorum* | 10–30 points | *Senecio nemorensis* | 10–30 points | *Tetraena  mongolica* | List of Key Protected Wild Plants in Inner Mongolia Autonomous Region |
| *Cardamine prorepens* | 10–30 points | *Ixeridium graminifolium* | 10–30 points | *Senecio vulgaris* | 10–30 points | *Trollius chinensis* | List of Key Protected Wild Plants in Inner Mongolia Autonomous Region |
| *Carduus crispus* | 10–30 points | *Juncus bufonius* | 10–30 points | *Serratula coronata* | 10–30 points | *Trollius ledebouri* | List of Key Protected Wild Plants in Inner Mongolia Autonomous Region |
| *Carex duriuscula* | 10–30 points | *Juniperus rigida* | 10–30 points | *Setaria arenaria* | 10–30 points | *Phellodendron amurense* | List of National Key Protected Wild Plants in China, List of Key Protected Wild Plants in Inner Mongolia Autonomous Region |
| *Carum buriaticum* | 10–30 points | *Kalidium gracile* | 10–30 points | *Sibbaldia adpressa* | 10–30 points | *Ranunculus japonicus* | the Convention on International Trade in Endangered Species of Wild Fauna and Flora |
| *Carum carvi* | 10–30 points | *Kalimeris indica* | 10–30 points | *Silene jenisseensis* | 10–30 points | *Gymnadenia conopsea* | the Convention on International Trade in Endangered Species of Wild Fauna and Flora, List of Key Protected Wild Plants in Inner Mongolia Autonomous Region |
| *Celosia cristata* | 10–30 points | *Kalimeris integrifolia* | 10–30 points | *Silene venosa* | 10–30 points | *Forsythia suspensa* | Wild herbal resources protection management regulations |
| *Centaurea cyanus* | 10–30 points | *Kochia scoparia* | 10–30 points | *Sisymbrium heteromallum* | 10–30 points | *Lithospermum erythrorhizon* | Wild herbal resources protection management regulations |
| *Cerastium arvense* | 10–30 points | *Kummerowia striata* | 10–30 points | *Sium suave* | 10–30 points | *Polygala tenuifolia* | Wild herbal resources protection management regulations |
| *Cerasus humilis* | 10–30 points | *Lagochilus ilicifolius* | 10–30 points | *Smilacina dahurica* | 10–30 points | *Cistanche deserticola* | Wild herbal resources protection management regulations, List of Key Protected Wild Plants in Inner Mongolia Autonomous Region |
| *Ceratoides arborescens* | 10–30 points | *Lamium album* | 10–30 points | *Solanum melongena* | 10–30 points | *Gentiana macrophylla* | Wild herbal resources protection management regulations, List of Key Protected Wild Plants in Inner Mongolia Autonomous Region |
| *Ceratoides latens* | 10–30 points | *Larix gmelinii* | 10–30 points | *Solanum septemlobum* | 10–30 points | *Gentiana scabra* | Wild herbal resources protection management regulations, List of Key Protected Wild Plants in Inner Mongolia Autonomous Region |
| *Chamaerhodos canescens* | 10–30 points | *Larix principis-rupprechtii* | 10–30 points | *Solanum tuberosum* | 10–30 points | *Glycyrrhiza uralensis* | Wild herbal resources protection management regulations, List of Key Protected Wild Plants in Inner Mongolia Autonomous Region |
| *Chamaerhodos trifida* | 10–30 points | *Lathyrus humilis* | 10–30 points | *Sophora japonica* | 10–30 points | *Scutellaria baicalensis* | Wild herbal resources protection management regulations, List of Key Protected Wild Plants in Inner Mongolia Autonomous Region |
| *Chelonopsis pseudobracteata* | 10–30 points | *Leontopodium conglobatum* | 10–30 points | *Sorbaria kirilowii* | 10–30 points | *Saposhnikovia divaricata* | Wild herbal resources protection management regulations、List of Key Protected Wild Plants in Inner Mongolia Autonomous Region |
| *Chenopodium acuminatum* | 10–30 points | *Lespedeza juncea* | 10–30 points | *Sorbus pohuashanensis* | 10–30 points | *Adenophora pereskiifolia* | more than 200 points |
| *Chenopodium foetidum* | 10–30 points | *Ligularia mongolica* | 10–30 points | *Sorghum bicolor* | 10–30 points | *Artemisia desertorum* | more than 200 points |
| *Chenopodium rubrum* | 10–30 points | *Ligularia sagitta* | 10–30 points | *Sparganium stoloniferum* | 10–30 points | *Artemisia frigida* | more than 200 points |
| *Chenopodium serotinum* | 10–30 points | *Ligularia sibirica* | 10–30 points | *Speranskia tuberculata* | 10–30 points | *Aster altaicus* | more than 200 points |
| *Chloris virgata* | 10–30 points | *Ligustrum quihoui* | 10–30 points | *Sphallerocarpus gracilis* | 10–30 points | *Atractylodes lancea* | more than 200 points |
| *Cicuta virosa* | 10–30 points | *Lilium dauricum* | 10–30 points | *Spiraea aquilegifolia* | 10–30 points | *Bupleurum chinense* | more than 200 points |
| *Cimicifuga foetida* | 10–30 points | *Lilium pumilum* | 10–30 points | *Stellaria cherleriae* | 10–30 points | *Bupleurum scorzonerifolium* | more than 200 points |
| *Cimicifuga simplex* | 10–30 points | *Limonium aureum* | 10–30 points | *Stellaria media* | 10–30 points | *Cannabis sativa* | more than 200 points |
| *Cirsium arvense* | 10–30 points | *Limonium tenellum* | 10–30 points | *Stipa capillata* | 10–30 points | *Caragana microphylla* | more than 200 points |
| *Cirsium esculentum* | 10–30 points | *Linaria buriatica* | 10–30 points | *Suaeda corniculata* | 10–30 points | *Clematis hexapetala* | more than 200 points |
| *Cirsium japonicum* | 10–30 points | *Lomatogonium micranthum* | 10–30 points | *Syneilesis aconitifolia* | 10–30 points | *Convallaria majalis* | more than 200 points |
| *Cirsium vlassovianum* | 10–30 points | *Lonicera chrysantha* | 10–30 points | *Syringa pubescens* | 10–30 points | *Cynanchum thesioides* | more than 200 points |
| *Cistanche deserticola* | 10–30 points | *Lonicera maackii* | 10–30 points | *Syringa reticulata* | 10–30 points | *Dianthus chinensis* | more than 200 points |
| *Citrullus lanatus* | 10–30 points | *Lonicera microphylla* | 10–30 points | *Tagetes erecta* | 10–30 points | *Dictamnus dasycarpus* | more than 200 points |
| *Clematis aethusifolia* | 10–30 points | *Lychnis sibirica* | 10–30 points | *Tagetes patula* | 10–30 points | *Echinops sphaerocephalus* | more than 200 points |
| *Clematis fruticosa* | 10–30 points | *Lycium chinense* | 10–30 points | *Tamarix ramosissima* | 10–30 points | *Fragaria orientalis* | more than 200 points |
| *Clematis macropetala* | 10–30 points | *Lycium truncatum* | 10–30 points | *Taraxacum borealisinense* | 10–30 points | *Galium verum* | more than 200 points |
| *Cleome spinosa* | 10–30 points | *Lycopersicon esculentum* | 10–30 points | *Taraxacum brassicaefolium* | 10–30 points | *Gentiana dahurica* | more than 200 points |
| *Commelina communis* | 10–30 points | *Lycopus lucidus* | 10–30 points | *Taraxacum dissectum* | 10–30 points | *Glycyrrhiza uralensis* | more than 200 points |
| *Convolvulus tragacanthoides* | 10–30 points | *Lysimachia davurica* | 10–30 points | *Taraxacum leucanthum* | 10–30 points | *Klasea centauroides* | more than 200 points |
| *Conyza canadensis* | 10–30 points | *Malus prunifolia* | 10–30 points | *Taraxacum ohwianum* | 10–30 points | *Lappula myosotis* | more than 200 points |
| *Corallodiscus flabellatus* | 10–30 points | *Malva sinensis* | 10–30 points | *Tephroseris flammea* | 10–30 points | *Leontopodium leontopodioides* | more than 200 points |
| *Coreopsis tinctoria* | 10–30 points | *Mazus stachydifolius* | 10–30 points | *Tephroseris kirilowii* | 10–30 points | *Lepidium apetalum* | more than 200 points |
| *Coriandrum sativum* | 10–30 points | *Melica scabrosa* | 10–30 points | *Thalictrum foetidum* | 10–30 points | *Lespedeza bicolor* | more than 200 points |
| *Corispermum declinatum* | 10–30 points | *Melilotus albus* | 10–30 points | *Thalictrum petaloideum* | 10–30 points | *Lilium pumilum* | more than 200 points |
| *Corispermum mongolicum* | 10–30 points | *Menispermum dauricum* | 10–30 points | *Thalictrum simplex* | 10–30 points | *Medicago ruthenica* | more than 200 points |
| *Corispermum stauntonii* | 10–30 points | *Metaplexis japonica* | 10–30 points | *Thesium longifolium* | 10–30 points | *Oxytropis myriophylla* | more than 200 points |
| *Corydalis adunca* | 10–30 points | *Mirabilis jalapa* | 10–30 points | *Thesium refractum* | 10–30 points | *Paeonia lactiflora* | more than 200 points |
| *Corydalis edulis* | 10–30 points | *Moehringia lateriflora* | 10–30 points | *Thlaspi thlaspidioides* | 10–30 points | *Parthenocissus tricuspidata* | more than 200 points |
| *Corylus heterophylla* | 10–30 points | *Morus alba* | 10–30 points | *Thymus quinquecostatus* | 10–30 points | *Plantago asiatica* | more than 200 points |
| *Cotoneaster integerrimus* | 10–30 points | *Myosotis silvatica* | 10–30 points | *Thymus serpyllum* | 10–30 points | *Plantago depressa* | more than 200 points |
| *Cotoneaster melanocarpus* | 10–30 points | *Myricaria bracteata* | 10–30 points | *Torularia humilis* | 10–30 points | *Polygala tenuifolia* | more than 200 points |
| *Cotoneaster soongoricus* | 10–30 points | *Nepeta cataria* | 10–30 points | *Triglochin maritimum* | 10–30 points | *Polygonatum odoratum* | more than 200 points |
| *Crataegus dahurica* | 10–30 points | *Nitraria sibirica* | 10–30 points | *Trigonotis peduncularis* | 10–30 points | *Polygonum aviculare* | more than 200 points |
| *Crataegus pinnatifida* | 10–30 points | *Oenothera biennis* | 10–30 points | *Tripolium vulgare* | 10–30 points | *Polygonum divaricatum* | more than 200 points |
| *Crataegus sanguinea* | 10–30 points | *Oxytropis bicolor* | 10–30 points | *Triticum aestivum* | 10–30 points | *Potentilla bifurca* | more than 200 points |
| *Cucumis melo* | 10–30 points | *Oxytropis ciliata* | 10–30 points | *Trollius chinensis* | 10–30 points | *Potentilla chinensis* | more than 200 points |
| *Cucumis sativus* | 10–30 points | *Oxytropis glabra* | 10–30 points | *Typha angustifolia* | 10–30 points | *Rhaponticum uniflorum* | more than 200 points |
| *Cucurbita moschata* | 10–30 points | *Oxytropis grandiflora* | 10–30 points | *Typha minima* | 10–30 points | *Sanguisorba officinalis* | more than 200 points |
| *Cuscuta japonica* | 10–30 points | *Oxytropis leptophylla* | 10–30 points | *Typha orientalis* | 10–30 points | *Saposhnikovia divaricata* | more than 200 points |
| *Cymbaria mongolica* | 10–30 points | *Oxytropis ochrantha* | 10–30 points | *Ulmus pumila* | 10–30 points | *Scutellaria baicalensis* | more than 200 points |
| *Cymbopogon distans* | 10–30 points | *Oxytropis ochrocephala* | 10–30 points | *Urtica angustifolia* | 10–30 points | *Scutellaria scordifolia* | more than 200 points |
| *Cynanchum hancockianum* | 10–30 points | *Panicum miliaceum* | 10–30 points | *Urtica fissa* | 10–30 points | *Stellera chamaejasme* | more than 200 points |
| *Cynanchum paniculatum* | 10–30 points | *Panzeria alaschanica* | 10–30 points | *Vaccinium uliginosum* | 10–30 points | *Taraxacum mongolicum* | more than 200 points |
| *Cypripedium guttatum* | 10–30 points | *Parasenecio hastatus* | 10–30 points | *Veratrum dahuricum* | 10–30 points | *Tribulus terrestris* | more than 200 points |
| *Cypripedium macranthum* | 10–30 points | *Parthenocissus quinquefolia* | 10–30 points | *Veronica anagallis-aquatica* | 10–30 points | *Vicia amoena* | more than 200 points |
| *Dendranthema naktongense* | 10–30 points | *Patrinia rupestris* | 10–30 points | *Veronica dahurica* | 10–30 points | *Xanthium strumarium* | more than 200 points |
| *Dendranthema zawadskii* | 10–30 points | *Pedicularis spicata* | 10–30 points | *Veronica didyma* | 10–30 points |  |  |
| *Dianthus chinensis* | 10–30 points | *Pedicularis verticillata* | 10–30 points | *Veronica longifolia* | 10–30 points |  |  |
| *Dianthus repens* | 10–30 points | *Peganum multisectum* | 10–30 points | *Viburnum opulus* | 10–30 points |  |  |

**Supplementary Table 4.** Model fitting results of relationship between ecological data and medicinal plant diversity

| Principal Components | edf | P-value |
| --- | --- | --- |
| s(PC1) | 1.903 | 0.000208 *** |
| s(PC2) | 1.000 | 0.000257 *** |
| s(PC3) | 1.922 | 0.000762 *** |
| s(PC4) | 1.000 | 0.227865 |

*** *P* < 0.001

**Supplementary Table 5.** Model fitting results of relationship between ecological data and medicinal plant diversity

| Principal Components | edf | P-value |
| --- | --- | --- |
| s(PC1) | 1.543 | 0.00575 ** |
| s(PC2) | 2.000 | 0.00417 ** |
| s(PC3) | 1.992 | 0.01096 * |
| s(PC4) | 2.000 | 0.00755 ** |
| s(PC5) | 1.642 | 0.00392 ** |

* *P* < 0.05, ** *P* < 0.01

# Supplementary Method

The field survey of this study has been making field survey methods since December 2012. Traditional survey methods were used as the basis, combined with modern technology such as GPS and GIS to ensure the accuracy of data in the survey. Through pre-discussions among experts of our subject group, we referred to the "Flora of Inner Mongolia", "Medicinal Flora of Inner Mongolia", "List of the Third Survey of Traditional Chinese Medicine Resources in Inner Mongolia", "Flora of China", "Higher Plants of China", "Chinese Materia Medica", "Chinese Traditional Chinese Medicine Resources", "Chinese Dao-Di Materia Medica", "Usage and Dosage of Commonly Used Traditional Chinese Medicine" and the list of rare and endangered plants, and initially formulated the list of the survey of medicinal plant resources in Inner Mongolia The survey will be carried out systematically in the whole region by means of route survey and sample survey, taking the county-level administrative unit of Inner Mongolia as the survey unit.

When conducting the route survey, after the botanical experts identified the medicinal plant species on site, their distribution latitude and longitude information and elevation information were recorded, while individual, community and habitat photos of wild medicinal plants were taken. Each wild medicinal plant resource appears at most five times on the same day or in the same township in the survey, and the interval is kept above 1km. The line survey can better grasp the distribution profile of medicinal plant resources, and a comprehensive understanding of the climate, topography, vegetation types, and soil types in the areas where medicinal plant resources are distributed will also be developed.

At the same time, by pre-confirming with the visit survey and market survey on different areas of the whole region set up a sample, based on the size of each survey unit, determine the number of sample plots in the representative area, so that the sample plots are evenly distributed within the county, for counties with larger areas, the number of survey sample plots should be appropriately increased to reduce the sampling error, while counties with smaller areas should appropriately reduce the number of sample plots set. Ultimately, each county to ensure that at least 3 to 6 sample sites are selected. If a site is not accessible during the field survey, a nearby sample site that meets the ecological characteristics of the table area can be selected for replacement. When conducting the sample survey, the GPS positioning system is supplemented with a GIS system to implement a view of the researcher's location. The above sample survey method was used to conduct a focused survey for possible key medicinal plant distribution areas, and information on medicinal plant resources in each sample was recorded. The original record data of the field survey are currently stored in the 3. Inner Mongolia Hospital of Traditional Chinese Medicine. Some of the non-recorded forms are shown in Figure 1.


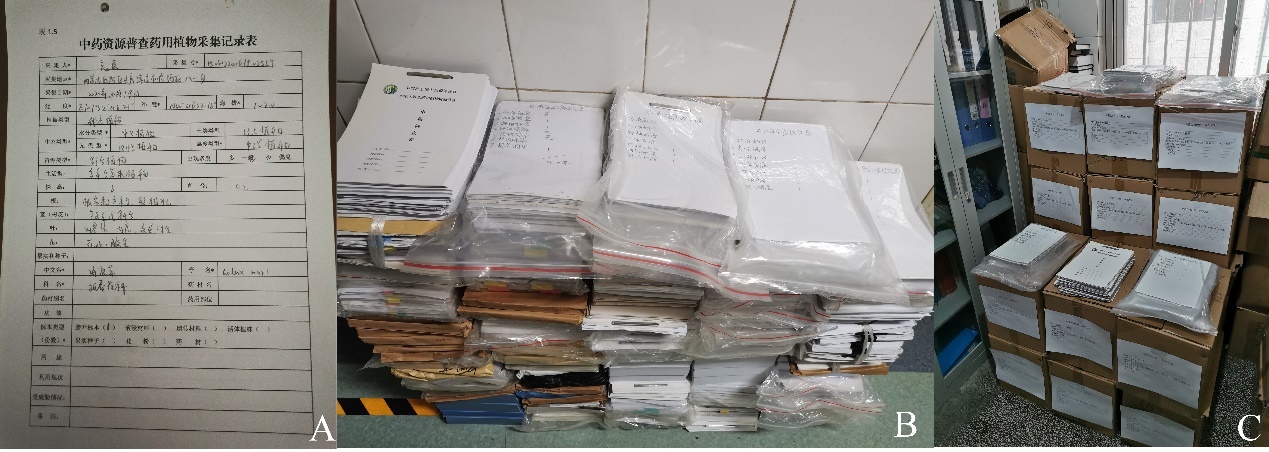


**Figure 1.** Original records of medicinal plant resources survey (A) and Storage of paper-based materials (B and C)

The survey work conducted by this group was divided into four time periods. Due to problems such as unreasonable sampling in the initial survey, field surveys were conducted in 34 banner counties in Inner Mongolia between 2012 and 2017; surveys were conducted in three batches in 2018, 2019 and 2020 for 15, 40 and 14 banner counties, respectively, and in 2021 to check the gaps and make up for some closing work. The detailed survey areas are shown in Figure 2. Figure 3 shows the photos of different areas during the route survey and sample survey conducted by the subject members.

In the end, we conducted 3938 sample plots for 103 banners and counties, completed 17,782 sample sets, conducted a perfect line survey, and investigated a total of 2,194 wild species of census, involving 140 families and 733 genera, among which 2 new species of *Rehmannia chrysantha* M. H. Li & C. H. Zhang. and *Euphorbia mongoliensis*. M. H. Li & C. H. Zhang were found^1,2^


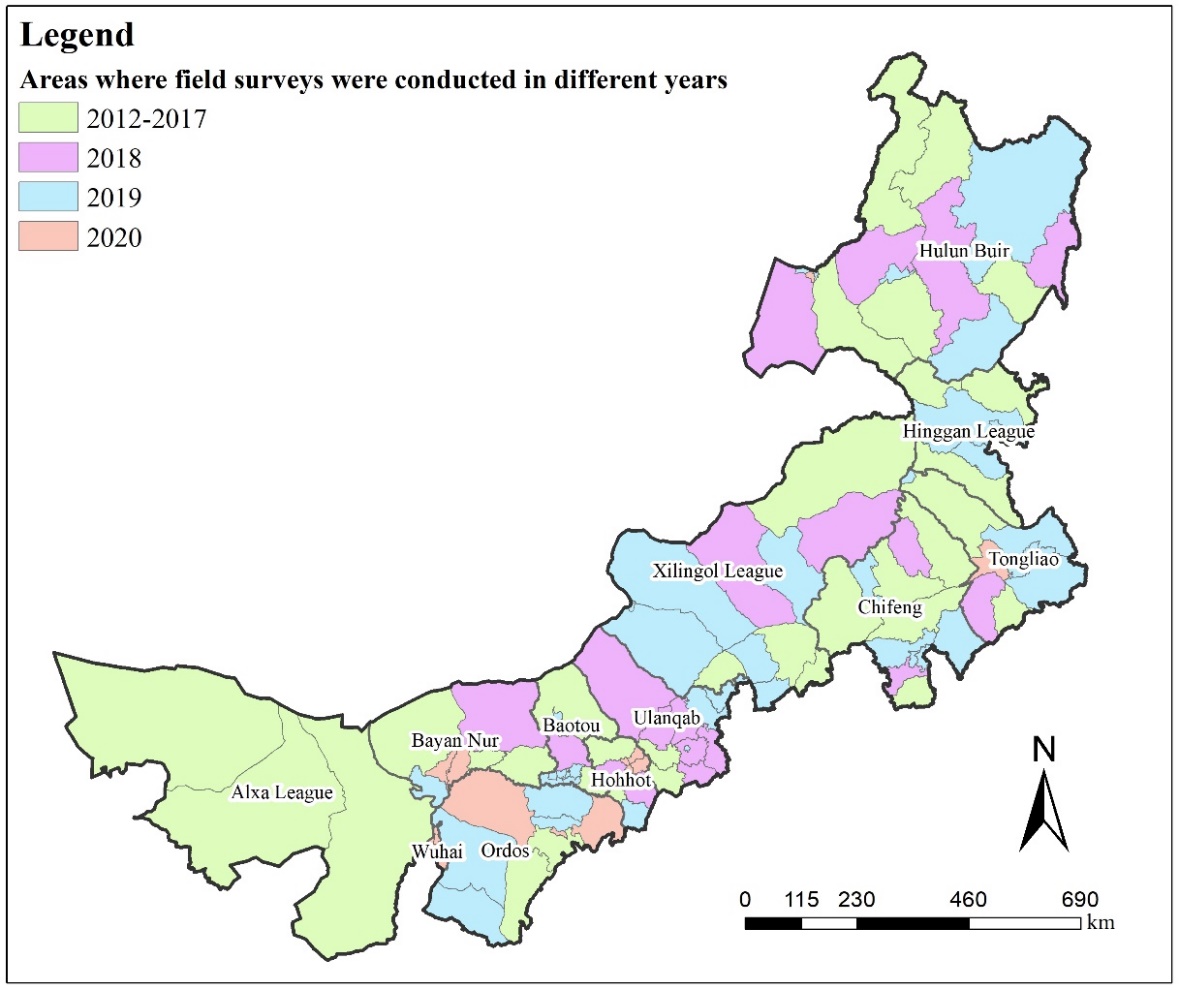


Figure 2 Display of years in which surveys were conducted in different regions


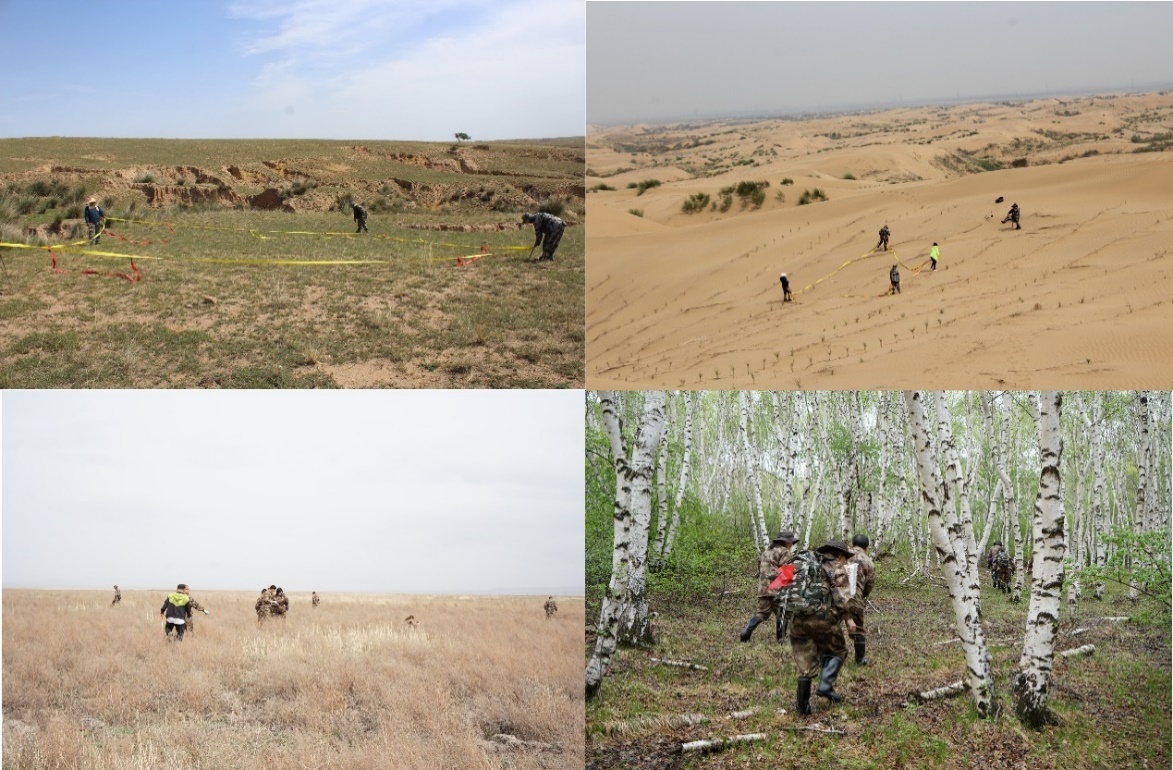


Figure 3 Research records of subject members during investigation

# Supplementary Result

## Analysis of the drivers influencing the richness of MPD

### Relationship between ecological data and medicinal plant diversity

We performed GAM analysis for all four spatial grids. We found that the model fits best with a spatial grid of 15 km. After principal component (PC) analysis, four PCs were selected with 89.86% of variables explained. The final GAM model fit R2 was 0.607, p<0.01, and the model explained 65.6% of the deviation. The reduced equations of the relationship between these four principal components and ecological factors are shown below, and they can demonstrate to some extent the effect of changes in these ecological factors on PC and further demonstrate the effect on MPD by GAM model fitting. The detailed analysis is shown in the body of the article.

[1]. PC1=-0.176 * bio_3 + 0.194 * bio_4-0.258 * bio_5-0.249 * bio_6 + 0.165 * bio_7-0.24 * bio_8-0.047 * bio_2 + 0.209 * bio_19-0.093 * elev-0.255 * bio_10-0.251 * bio_11 + 0.249 * bio_12 + 0.242 * bio_13 + 0.197 * bio_14 + 0.189 * bio_15 + 0.249 * bio_16 + 0.203 * bio_17-0.262 * bio_1 + 0.25 * bio_18-0.255 * bio_9 + 0.154 * Zblx-0.134 * Trlx

[2]. PC2=-0.33 * bio_3 + 0.378 * bio_4 + 0.107 * bio_5-0.191 * bio_6 + 0.38 * bio_7 + 0.161 * bio_8-0.005 * bio_2-0.277 * bio_19-0.288 * elev + 0.089 * bio_10-0.195 * bio_11-0.134 * bio_12-0.107 * bio_13-0.264 * bio_14 + 0.129 * bio_15-0.102 * bio_16-0.303 * bio_17-0.095 * bio_1-0.096 * bio_18-0.17 * bio_9-0.239 * Zblx + 0.034 * Trlx

[3]. PC3=-0.054 * bio_3 + 0.115 * bio_4-0.07 * bio_5-0.156 * bio_6 + 0.18 * bio_7-0.16 * bio_8 + 0.122 * bio_2 + 0.177 * bio_19 + 0.413 * elev-0.156 * bio_10-0.154 * bio_11-0.219 * bio_12-0.286 * bio_13 + 0.208 * bio_14-0.346 * bio_15-0.25 * bio_16 + 0.168 * bio_17-0.164 * bio_1-0.246 * bio_18-0.115 * bio_9-0.078 * Zblx + 0.277 * Trlx

[4]. PC4=0.355 * bio_3 + 0.053 * bio_4 + 0.124 * bio_5-0.104 * bio_6 + 0.263 * bio_7 + 0.026 * bio_8 + 0.786 * bio_2 + 0.005 * bio_19-0.159 * elev + 0.031 * bio_10-0.013 * bio_11 + 0.041 * bio_12 + 0.033 * bio_13-0.038 * bio_14-0.209 * bio_15 + 0.032 * bio_16 + 0.011 * bio_17 + 0.001 * bio_1 + 0.031 * bio_18-0.001 * bio_9 + 0.179 * Zblx-0.222 * Trlx

### Relationship between human social development data and medicinal plant diversity

We collected different data when using counties and cities as statistical units, and we performed separate GAM analyses. The results of the analysis based on county-level data are as follows.

Through principal component analysis, we found that the cumulative proportion reached 90.34% when the number of principal components (PC) was 10, so we selected the first 10 PCs with MPD for GAM analysis, The relationship between each PC and socio-economic factors is as follows.

[1] PC1=-0.015 * NVTCHM + 0.025 * TKCM + 0.242 * TPCH + 0.191 * CACHM + 0.158 * NHCS + 0.263 * NCHMCE + 0.23 * NCMPPE + 0.338 * NCMWE + 0.336 * NCMRE + 0.234 * NCHMSE + 0.338 * Population + 0.144 * TLA-0.071 * CA + 0.078 * RM + 0.337 * RP + 0.292 * RWM + 0.276 * RCHM + 0.232 * RPCM"

[2] PC2=0.155 * NVTCHM + 0.091 * TKCM + 0.324 * TPCH + 0.291 * CACHM + 0.084 * NHCS + 0.034 * NCHMCE + 0.227 * NCMPPE + 0.307 * NCMWE + 0.314 * NCMRE + 0.087 * NCHMSE-0.064 * Population-0.149 * TLA-0.057 * CA-0.152 * RM-0.392 * RP-0.358 * RWM-0.254 * RCHM-0.345 * RPCM

[3] PC3=-0.099 * NVTCHM + 0.096 * TKCM + 0.246 * TPCH + 0.321 * CACHM + 0.255 * NHCS + 0.348 * NCHMCE-0.201 * NCMPPE-0.074 * NCMWE-0.083 * NCMRE-0.319 * NCHMSE-0.25 * Population + 0.238 * TLA + 0.388 * CA + 0.398 * RM-0.034 * RP + 0.078 * RWM-0.197 * RCHM + 0.03 * RPCM

[4] PC4=-0.547 * NVTCHM-0.496 * TKCM-0.174 * TPCH + 0.022 * CACHM-0.469 * NHCS + 0.176 * NCHMCE + 0.052 * NCMPPE + 0.22 * NCMWE + 0.215 * NCMRE-0.041 * NCHMSE + 0.027 * Population + 0.063 * TLA + 0.205 * CA + 0.063 * RM-0.091 * RP-0.097 * RWM-0.062 * RCHM-0.046 * RPCM

[5] PC5=0.004 * NVTCHM + 0.149 * TKCM + 0.085 * TPCH + 0.291 * CACHM-0.275 * NHCS-0.106 * NCHMCE-0.228 * NCMPPE + 0.142 * NCMWE + 0.141 * NCMRE-0.171 * NCHMSE-0.282 * Population-0.595 * TLA + 0.135 * CA-0.231 * RM + 0.189 * RP + 0.175 * RWM + 0.026 * RCHM + 0.325 * RPCM

[6] PC6=0.015 * NVTCHM-0.566 * TKCM + 0.325 * TPCH + 0.172 * CACHM + 0.145 * NHCS + 0.114 * NCHMCE-0.19 * NCMPPE-0.128 * NCMWE-0.126 * NCMRE-0.319 * NCHMSE + 0 * Population + 0.069 * TLA-0.451 * CA-0.351 * RM + 0.037 * RP-0.011 * RWM + 0.083 * RCHM + 0.029 * RPCM

[7] PC7=-0.424 * NVTCHM + 0.012 * TKCM + 0.069 * TPCH + 0.21 * CACHM + 0.248 * NHCS + 0.146 * NCHMCE + 0.238 * NCMPPE-0.323 * NCMWE-0.321 * NCMRE + 0.388 * NCHMSE-0.033 * Population-0.242 * TLA + 0.258 * CA-0.298 * RM + 0.016 * RP-0.109 * RWM + 0.21 * RCHM-0.056 * RPCM

[8] PC8=0.303 * NVTCHM-0.295 * TKCM + 0.098 * TPCH + 0.169 * CACHM-0.018 * NHCS-0.244 * NCHMCE-0.254 * NCMPPE-0.017 * NCMWE-0.034 * NCMRE + 0.013 * NCHMSE + 0.347 * Population-0.202 * TLA + 0.369 * CA + 0.293 * RM + 0.015 * RP-0.192 * RWM + 0.424 * RCHM-0.241 * RPCM

[9] PC9=-0.453 * NVTCHM-0.039 * TKCM + 0.183 * TPCH-0.096 * CACHM + 0.3 * NHCS-0.543 * NCHMCE + 0.187 * NCMPPE + 0.087 * NCMWE + 0.088 * NCMRE-0.179 * NCHMSE-0.185 * Population-0.173 * TLA-0.21 * CA + 0.362 * RM + 0.065 * RP + 0.108 * RWM + 0.092 * RCHM-0.137 * RPCM

[10] PC10=0.217 * NVTCHM-0.336 * TKCM-0.012 * TPCH-0.076 * CACHM + 0.178 * NHCS-0.116 * NCHMCE + 0.483 * NCMPPE-0.088 * NCMWE-0.062 * NCMRE-0.174 * NCHMSE + 0.241 * Population-0.213 * TLA + 0.276 * CA + 0.018 * RM-0.043 * RP + 0.078 * RWM-0.425 * RCHM + 0.381 * RPCM

The results of the analysis showed that the R^2^ of the GAM model was 0.249 and the explained deviation reached 65.6% (Figure 4). Therefore, the results of the GAM analysis at the county level were not satisfactory, which may be related to the expansion of the data volume and the limitations that exist in data collection. Also, the socioeconomic complexity may be one of the factors contributing to such results.

**Table 1**. Model fitting results of relationship between ecological data and medicinal plant diversity at county-level

| Principal Components | edf | P-value |
| --- | --- | --- |
| s(PC1) | 1.000 | 0.37070 |
| s(PC2) | 1.000 | 0.65076 |
| s(PC3) | 1.441 | 0.00281** |
| s(PC4) | 1.816 | 0.00299 ** |
| s(PC5) | 1.00 | 0.68634 |
| s(PC6) | 1.00 | 0.53994 |
| s(PC7) | 1.00 | 0.26281 |
| s(PC8) | 1.00 | 0.84312 |
| s(PC9) | 1.595 | 0.46542 |
| s(PC10) | 1.821 | 0.12522 |

** *P* < 0.01


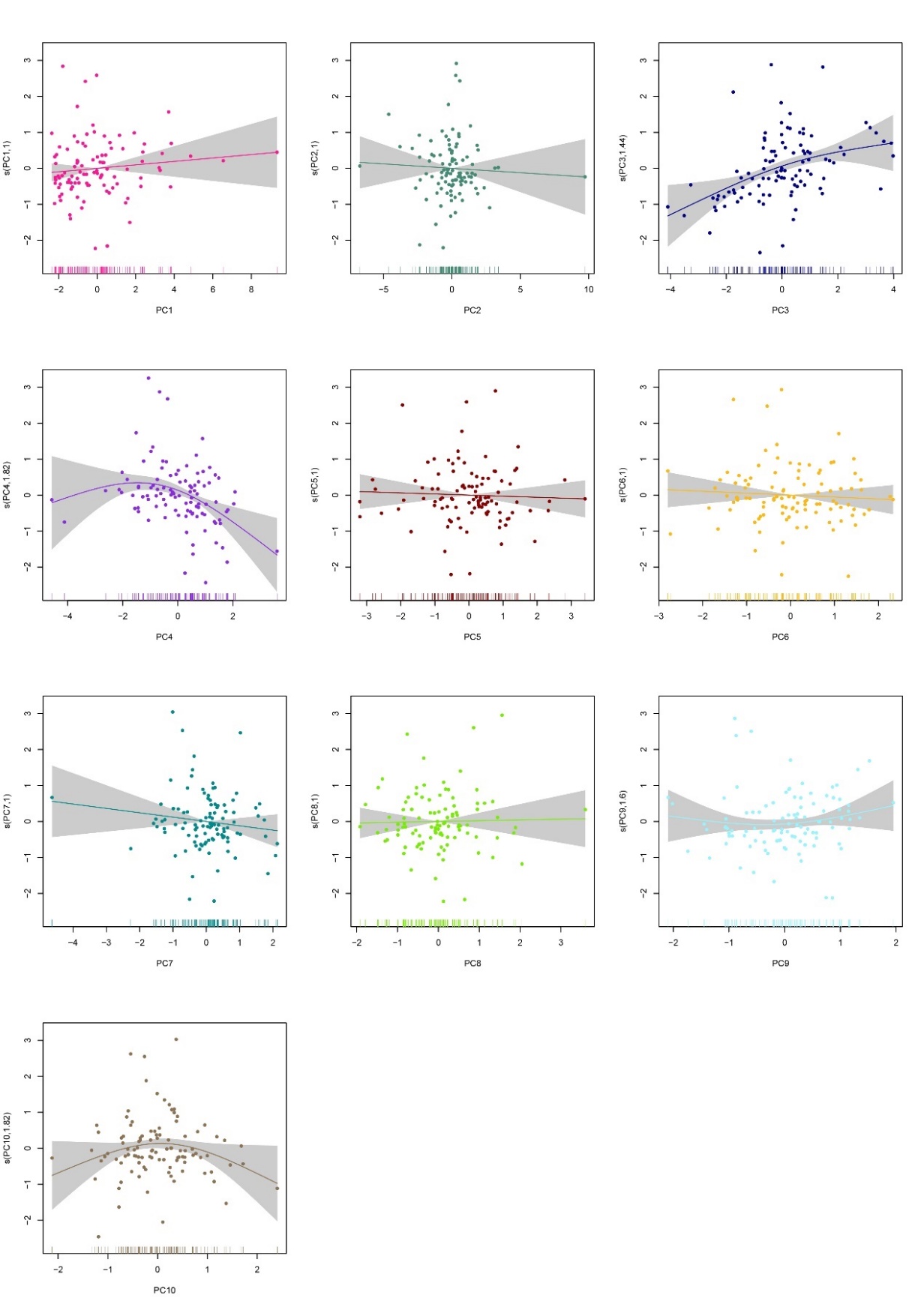


**Figure 4.** Relationship between each variable and medicinal plants diversity after principal component analysis at county-level

At city-level, the 25 influential factors were reduced to 5 (cumulative contribution was 89.63%) by PC analysis, with R2 of 0.996, P < 0.01. The relevant results are presented in the body of the article, and in the supplementary material we present the relationship between PC and various socioeconomic factors.

[1] PC1=-0.256 * GPBMHE-0.055 * GDPSI-0.106 * GDPTI-0.25 * GDPPI-0.111 * GDP-0.207 * TLA-0.232 * NHI-0.238 * NHFP-0.2 * CA-0.033 * NVTCHM-0.189 * TKCM-0.211 * TPCH-0.103 * CACHM-0.236 * NHCS-0.193 * NCHMCE-0.2 * NCMPPE-0.214 * NCMWE-0.203 * NCMRE-0.185 * NCHMSE-0.236 * Population-0.197 * RM-0.252 * RP-0.224 * RWM-0.238 * RCHM-0.202 * RPCM

[2] PC2=0.061 * GPBMHE + 0.32 * GDPSI + 0.38 * GDPTI-0.137 * GDPPI + 0.362 * GDP-0.204 * TLA + 0.064 * NHI + 0.179 * NHFP-0.207 * CA + 0.02 * NVTCHM-0.092 * TKCM + 0.001 * TPCH-0.087 * CACHM-0.033 * NHCS-0.282 * NCHMCE + 0.284 * NCMPPE + 0.147 * NCMWE + 0.176 * NCMRE + 0.264 * NCHMSE + 0.055 * Population-0.127 * RM-0.154 * RP-0.234 * RWM + 0.085 * RCHM-0.272 * RPCM

[3] PC3=0.203 * GPBMHE + 0.277 * GDPSI + 0.182 * GDPTI + 0.021 * GDPPI + 0.246 * GDP + 0.133 * TLA-0.049 * NHI + 0.025 * NHFP-0.098 * CA + 0.392 * NVTCHM + 0.192 * TKCM-0.267 * TPCH-0.499 * CACHM + 0.051 * NHCS + 0.103 * NCHMCE-0.154 * NCMPPE-0.242 * NCMWE-0.248 * NCMRE-0.176 * NCHMSE + 0.034 * Population + 0.16 * RM + 0.035 * RP + 0.067 * RWM-0.087 * RCHM + 0.135 * RPCM

[4] PC4=0.019 * GPBMHE-0.247 * GDPSI-0.187 * GDPTI + 0.163 * GDPPI-0.216 * GDP + 0.107 * TLA + 0.349 * NHI + 0.016 * NHFP-0.34 * CA + 0.426 * NVTCHM + 0.039 * TKCM + 0.305 * TPCH-0.04 * CACHM + 0.283 * NHCS-0.24 * NCHMCE + 0.043 * NCMPPE-0.148 * NCMWE-0.145 * NCMRE + 0.063 * NCHMSE + 0.154 * Population-0.014 * RM-0.148 * RP-0.18 * RWM-0.021 * RCHM-0.197 * RPCM

[5] PC5=-0.058 * GPBMHE-0.267 * GDPSI + 0.15 * GDPTI + 0.065 * GDPPI-0.05 * GDP + 0.255 * TLA + 0.095 * NHI + 0.303 * NHFP-0.053 * CA-0.174 * NVTCHM-0.388 * TKCM-0.119 * TPCH-0.257 * CACHM-0.18 * NHCS + 0.133 * NCHMCE + 0.143 * NCMPPE-0.197 * NCMWE-0.205 * NCMRE + 0.021 * NCHMSE + 0.204 * Population-0.457 * RM + 0.127 * RP + 0.079 * RWM + 0.201 * RCHM + 0.049 * RPCM
